# Mitochondrial metabolic regulation of macrophage polarization in osteomyelitis and other orthopedic disorders: mechanisms and therapeutic opportunities

**DOI:** 10.3389/fcell.2025.1604320

**Published:** 2025-06-13

**Authors:** Jinglin Li, Lin Zhang, Jiaze Peng, Chuntao Zhao, Wenguang Li, Yang Yu, Xianpeng Huang, Fuyin Yang, Xuan Deng, Xuxu Yang, Tao Zhang, Jiachen Peng

**Affiliations:** ^1^ Department of Orthopedics, Affiliated Hospital of Zunyi Medical University, Zunyi, China; ^2^ Department of Orthopedic Surgery, 920th Hospital of Joint Logistics Support Force, Kunming, China; ^3^ Zhejiang Provincial People’s Hospital Bijie Hospital, Bijie, China; ^4^ Key Laboratory of Cell Engineering of Guizhou Province, Affiliated Hospital of Zunyi Medical University, Zunyi, China; ^5^ Joint Orthopaedic Research Center of Zunyi Medical University, University of Rochester Medical Center, Zunyi, China

**Keywords:** mitochondrial metabolism, macrophage polarization, osteomyelitis, mitochondrial dynamics, inflammation, bone repair

## Abstract

Osteomyelitis is a complex infectious bone disease involving pathogen invasion, host immune responses, and dysregulation of the local microenvironment. As a critical component of the innate immune system, macrophages play a pivotal role in inflammatory responses and tissue repair. Their polarization states (M1/M2) directly influence disease progression, while mitochondrial metabolism, as the central hub of cellular energy metabolism, has recently been shown to play a key role in macrophage polarization and functional regulation. However, how mitochondrial metabolism regulates macrophage polarization to affect the pathological mechanisms of osteomyelitis, and how to develop novel therapeutic strategies based on this mechanism, remain critical scientific questions to be addressed. This review systematically summarizes the molecular mechanisms by which mitochondrial metabolism regulates macrophage polarization and its role in osteomyelitis, with a focus on the impact of mitochondrial dynamics (fission/fusion), metabolic reprogramming, and reactive oxygen species (ROS) generation on macrophage polarization. Additionally, potential therapeutic strategies targeting mitochondrial metabolism are analyzed. For the first time, this review integrates the interplay between mitochondrial metabolism and macrophage polarization in osteomyelitis, revealing how mitochondrial dysfunction exacerbates inflammation and bone destruction through metabolic reprogramming. Based on these findings, we propose novel therapeutic strategies targeting mitochondrial metabolism, offering new perspectives and directions for understanding the pathogenesis and clinical treatment of osteomyelitis.

## 1 Introduction

Osteomyelitis is a chronic inflammatory disease caused by pathogenic microbial invasion, characterized by the destruction of bone and surrounding soft tissues. The infection may be confined to a single bone or extend to multiple structures, including the bone marrow, cortical bone, periosteum, and adjacent soft tissues (Lew and Waldvogel, 2004). Among the primary pathogens, *Staphylococcus aureus* and *Staphylococcus epidermidis* are the most common (Kavanagh et al., 2018). *S. aureus* invades host tissues through intracellular infection, small colony variants (SCVs), biofilm formation, and abscess development. It also secretes various toxins that trigger local inflammatory responses, activating keratinocytes, T-helper cells, macrophages, neutrophils, and other immune cells (Masters et al., 2022; Chen H. et al., 2022). Globally, the incidence of osteomyelitis varies significantly by region. In the United States, the annual incidence is approximately 22 per 100,000 individuals (Kremers et al., 2015), with 10%–30% of acute cases progressing to chronic osteomyelitis (Wang X. et al., 2023).The rising incidence and emergence of antibiotic-resistant strains pose significant challenges to antibiotic therapy and surgical interventions, increasing clinical burden and healthcare costs.

Macrophages, as key components of the innate immune system, play a crucial role in immune responses during osteomyelitis by participating in tissue repair and homeostasis maintenance (Pidwill et al., 2020). These cells exhibit phagocytic and bactericidal functions and can polarize into pro-inflammatory M1 or anti-inflammatory M2 phenotypes in response to the infection microenvironment. M1 macrophages dominate the early phase of infection by recognizing pathogens, initiating inflammatory responses, secreting pro-inflammatory cytokines, activating endothelial cells, and recruiting immune cells such as neutrophils to the infection site. In contrast, M2 macrophages facilitate the resolution of inflammation in later stages by engulfing apoptotic cells, depositing collagen, and releasing anti-inflammatory mediators to promote tissue repair (Mills, 2015). Moreover, in the bone microenvironment, M1 macrophages serve as precursors for osteoclasts, differentiating into mature bone-resorbing cells, whereas M2 macrophages secrete osteogenic factors that stimulate mesenchymal stem cells to differentiate into osteoblasts (Hu et al., 2023), thereby facilitating bone regeneration. The imbalance of M1/M2 macrophage polarization in osteomyelitis not only exacerbates local inflammation but also disrupts bone homeostasis (Mouton et al., 2020). Due to the distinct pathological mechanisms of osteomyelitis, macrophages exhibit different functional states during the acute and chronic phases, leading to diverse clinical outcomes.

Mitochondria serve as central hubs for metabolic regulation in inflammatory and cellular stress responses, influencing macrophage polarization through mechanisms such as signal transduction, chromatin remodeling, and gene transcription regulation (Wang et al., 2021). The dynamic modulation of macrophage polarization and function by mitochondrial metabolism is critically involved in the pathogenesis of osteomyelitis (Dong et al., 2022). Previous studies have largely focused on the independent role of macrophage polarization or the isolated regulatory effects of mitochondrial metabolism in bone infections. However, the direct interplay between mitochondrial metabolism and macrophage polarization—and its impact on osteomyelitis progression—remains insufficiently explored. Given the challenges in osteomyelitis treatment, a deeper understanding of immune cell functional regulation is essential. This review integrates the cross-disciplinary mechanisms of mitochondrial metabolism and macrophage polarization for the first time, highlighting their synergistic roles in osteomyelitis. By elucidating the mitochondrial metabolic regulation of macrophage polarization, this work provides new insights and potential therapeutic strategies for osteomyelitis management.

## 2 Molecular mechanisms of mitochondrial metabolism in regulating Macrophage polarization

### 2.1 Metabolic characteristics of M1/M2 macrophage polarization

Macrophages, as key components of the immune system, play a crucial regulatory role in infection and inflammatory responses (Voll et al., 1997; Fadok et al., 1998; Ley et al., 2007).Upon stimulation by the infectious microenvironment, macrophages can polarize into either the pro-inflammatory M1 phenotype or the anti-inflammatory M2 phenotype (Wang et al., 2019; Xia et al., 2023).M1 macrophages exhibit a distinct “metabolic shift,” characterized by enhanced glycolysis, where hypoxia-inducible factor-1α (HIF-1α) upregulates glucose transporter 1 (GLUT1)and hexokinase 2 (HK2), driving rapid ATP and lactate production (Viola et al., 2019).Dysregulated tricarboxylic acid (TCA) cycle activity leads to abnormal accumulation of succinate and itaconate, directly influencing the pro-inflammatory function of M1 macrophages (Liu and Ho, 2018).Succinate stabilizes HIF-1α and promotes reactive oxygen species (ROS) production, forming a positive feedback loop that amplifies glycolysis and enhances pro-inflammatory gene expression (Van den Bossche et al., 2016; Tannahill et al., 2013). In contrast, M2 macrophages maintain an intact TCA cycle and rely primarily on oxidative phosphorylation (OXPHOS) for energy metabolism. Fatty acid oxidation (FAO) and glutamine metabolism serve as major ATP sources, supporting anti-inflammatory gene expression (Gobert and Wilson, 2012). Glutamine enhances M2 polarization by activating pyruvate dehydrogenase (PDH) (Zhu et al., 2022).Additionally, elevated FAO levels synergistically enhance lipid metabolism, further driving M2 phenotype formation (Li J. et al., 2024). Notably, a reduction in FAO activity may promote M1 polarization (Soliman et al., 2020). M1 activation suppresses mitochondrial OXPHOS while increasing nitric oxide (NO) production, effectively blocking the repolarization toward the M2 phenotype (Saxena et al., 2018).Moreover, hypoxic microenvironments induce HIF-1α accumulation, leading to mitochondrial protein lactylation modifications that significantly impair OXPHOS functionality, ultimately reducing M2 polarization efficiency (Mao et al., 2024).

### 2.2 M1/M2 Macrophage metabolic reprogramming and osteoclast differentiation

Osteoclasts arise from monocyte–macrophage precursors under the influence of receptor activator of nuclear factor κB ligand (RANKL) and macrophage colony-stimulating factor (M-CSF) (Qin et al., 2024). The distinct metabolic programs of M1 and M2 macrophages—through their differential regulation of energy pathways, signaling activity, and metabolite production—profoundly shape osteoclastogenesis and osteoclast function. M1 Macrophages rely predominantly on glycolysis, driven by HIF-1α signaling, and produce high levels of lactate and succinate (Viola et al., 2019; Liu and Ho, 2018). RANKL stimulation activates lactate dehydrogenase A (LDHA), further enhancing glycolysis and lactate output, which promotes osteoclast differentiation (Nishioku et al., 2023). Succinate also activates NF-κB in macrophages, amplifying both osteoclastogenesis and pro-inflammatory cytokine release (Guo et al., 2017). In a rheumatoid arthritis model, quercetin-laden nanoparticles inhibit the ERK/HIF-1α/GLUT1 axis, reduce M1 glycolysis, and skew macrophages toward an M2 phenotype—thereby curbing pro-inflammatory cytokines and restraining osteoclast overactivation (Jia et al., 2023). Moreover, M1-derived cytokines (e.g., TNF-α, IL-6) directly potentiate osteoclast activity. Pseudolaric acid B promotes M1→M2 repolarization and suppresses IL-1β, TNF-α, and IL-6 synthesis, while inhibiting RANKL-driven osteoclastogenesis via NF-κB and ERK pathway blockade (Liu L. et al., 2024). M2 Macrophages, by contrast, depend on OXPHOS and FAO. Activation of AMPK and PPARγ signaling in M2 cells inhibits osteoclast formation. For example, PPARγ dephosphorylation drives lipid synthesis and M2c polarization, leading to IL-10 secretion and tissue repair (Zuo et al., 2024). A lithographically fabricated forest-like silicon substrate has been shown to increase M2 markers, decrease inflammatory cytokines, and downregulate RANKL expression, thus restoring the balance between bone resorption and formation (Sun G. et al., 2024). Metabolic reprogramming can also be targeted to impede osteoclastogenesis: co-delivery of quercetin and catalase nanoparticles promotes M2 polarization, alleviates hypoxia, and suppresses osteoclast activity (Sun et al., 2025). Furthermore, mechanical cues—such as uniaxial compression applied via 3D-printed scaffolds—enhance M2 polarization, inhibit osteoclast formation, and accelerate bone repair (Kontogianni et al., 2025). In summary, M1/M2 macrophage metabolic reprogramming—via distinct energy pathways and associated signaling—modulates key metabolites and cytokine release to regulate osteoclast differentiation and function. Targeting macrophage polarization thus offers a promising strategy to suppress excessive bone resorption and restore bone homeostasis in inflammatory bone diseases (Table 1).

**TABLE 1:** Metabolic characteristics of macrophages and osteoclasts.

Cell type	Metabolic profile	Key Enzymes/Metabolites	Energy source	Regulatory pathways	Major products and functional impact
M1 Macrophages	Glycolysis-dominant (Warburg effect)	HIF-1α (stabilizes glycolytic genes), iNOS, HK2	Glucose (via glycolysis)	NF-κB, STAT1, HIF-1α	TNF-α, IL-6, IL-12, ROS, lactate, succinate → promotes osteoclast differentiation
M2 Macrophages	OXPHOS- and FAO-dominant	PPARγ (promotes FAO), Arg1, IL-10	Fatty acids, glutamine (via OXPHOS)	STAT6, AMPK, PPARγ	IL-10, TGF-β → inhibits osteoclast differentiation
Osteoclasts	Early differentiation relies on glycolysis; mature osteoclasts require mitochondrial respiration	TRAP, CTSK, NFATc1	Glucose, glutamine	RANKL/OPG signaling, MAPK/NF-κB	CTSK, H^+^ secretion (acidic microenvironment), ROS → drives bone resorption

### 2.3 Key signaling pathways mediating metabolic regulation

#### 2.3.1 HIF-1α-mediated pathway: driving M1 glycolysis and inflammation

**FIGURE 1:**
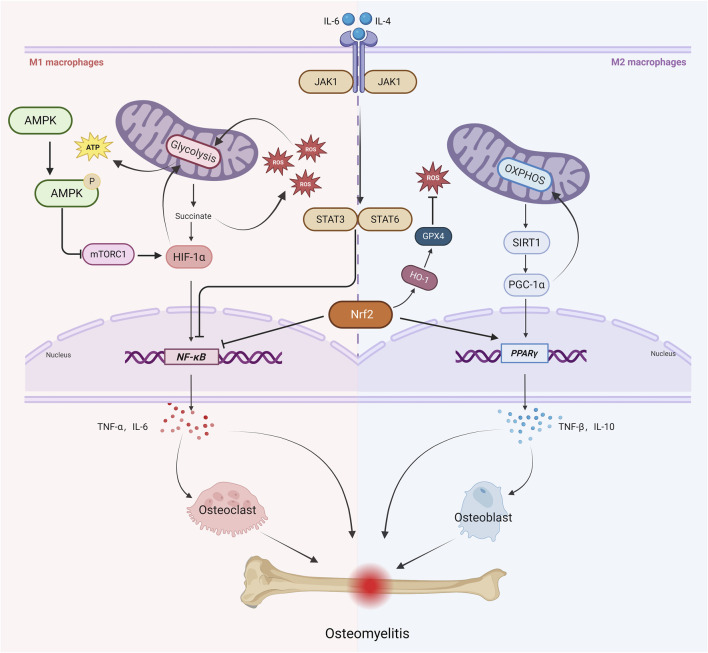
Key Signaling Pathways Linking Mitochondrial Metabolism, Macrophage Polarization, and Osteomyelitis Progression. Under infectious and inflammatory conditions, M1 macrophages primarily rely on glycolysis. Enhanced glycolysis leads to succinate accumulation, which promotes ROS production and further amplifies glycolysis through positive feedback and stabilizes HIF-1α and NF-κB signaling. This stabilization induces the secretion of pro-inflammatory cytokines such as TNF-α and IL-6, activating osteoclasts and exacerbating bone resorption. Meanwhile, mTORC1 promotes the upregulation of glycolysis-related genes by activating HIF-1α, reinforcing the pro-inflammatory feedback loop, whereas AMPK inhibits mTORC1 activity via phosphorylation. IL-6 activates the JAK1-STAT3 axis to enhance M1 polarization, whereas IL-4 promotes M2 polarization through the JAK1-STAT6 axis. Additionally, the JAK1/STAT3/6 pathway suppresses NF-κB signaling. M2-type macrophages primarily rely on oxidative phosphorylation (OXPHOS) for their energy metabolism. Enhanced OXPHOS activity increases the activity of SIRT1, which in turn activates PGC-1α. PGC-1α further enhances OXPHOS activity and regulates PPARγ activity, thereby promoting the secretion of anti-inflammatory cytokines such as IL-10 and TGF-β, ultimately facilitating osteogenesis and tissue repair. At the same time, Nrf2 activates the HO-1/GPX4 axis to reduce ROS levels and inhibit NF-κB signaling, thereby constraining M1 polarization, while simultaneously activating PPARγ to promote M2 polarization. These interconnected pathways orchestrate mitochondrial metabolism and macrophage polarization in osteomyelitis, ultimately dictating the balance between inflammatory bone destruction and reparative bone formation.

As a key regulatory molecule, hypoxia-inducible factor-1α (HIF-1α) orchestrates the metabolic reprogramming of glycolysis and the secretion of inflammatory factors, playing a pivotal role in M1 macrophage polarization. It is particularly crucial in infections, hypoxia, and chronic inflammatory diseases (Soto-Heredero et al., 2020). Under hypoxic or inflammatory stimuli (such as LPS and TNF-α), HIF-1α stability increases, driving the transcription of glycolysis-related genes (PKM2, HK2). This promotes aerobic glycolysis (the Warburg effect) while inhibiting OXPHOS, meeting the rapid energy demands of M1 macrophages (Viola et al., 2019).Notably, the upregulation of pyruvate dehydrogenase kinase1(PDK1)suppresses pyruvate dehydrogenase (PDH), preventing pyruvate entry into the tricarboxylic acid (TCA) cycle and further reinforcing the Warburg effect.Studies have shown that in M1-polarized macrophages, key glycolytic enzymes (GLUT1, HK2, LDHA, PKM2) and signaling proteins (Akt, HIF-1α, mTOR) are downregulated in mitochondria. Restoring OXPHOS function can facilitate macrophage repolarization from the M1 to the M2 phenotype (Fan et al., 2022). Moreover, TCA cycle disruption in M1 macrophages leads to succinate accumulation, which enhances inflammation through two mechanisms: stabilizing HIF-1α to upregulate pro-inflammatory cytokines such as IL-1β and TNF-α, and promoting mitochondrial reactive oxygen species (mtROS) production, which activates the NLRP3 inflammasome (Tannahill et al., 2013). Additionally, HIF-1α can form a positive feedback loop with the NF-κB signaling pathway, further amplifying pro-inflammatory cytokine expression and exacerbating chronic inflammation (Xu et al., 2022) (Figure 1).

#### 2.3.2 PGC-1α-mediated pathway: promoting M2 oxidative phosphorylation and tissue repair

Peroxisome proliferator-activated receptor gamma coactivator-1 alpha (PGC-1α) is a central regulator of OXPHOS and tissue repair in M2 macrophages. As a transcriptional coactivator, PGC-1α interacts with transcription factors such as peroxisome proliferator-activated receptor gamma (PPARγ) to activate downstream gene expression networks (Qian et al., 2024).Together, they upregulate carnitine palmitoyltransferase1A (CPT1A),facilitating the mitochondrial transport and β-oxidation of long-chain fatty acids, thereby promoting tissue repair. Conditional gene knockout mouse models and siRNA-mediated gene knockdown experiments shows that activation of the PGC-1α signaling pathway in alveolar macrophages significantly enhances mitochondrial OXPHOS activity, maintaining M2 immune phenotype stability. In contrast, PGC-1α deficiency weakens M2 functionality and delays the tissue repair process (Feng Z. et al., 2023). Notably, a hyperglycemic microenvironment inhibits PGC-1α expression, significantly impairing M2 polarization and tissue repair capacity (Zhao et al., 2023).Interventional studies suggest that PGC-1α overexpression can effectively mitigate inflammation and accelerate tissue regeneration (Chatsirisupachai et al., 2024). Mechanistically, PGC-1α sustains an anti-inflammatory microenvironment through dual pathways: it inhibits NF-κB signaling, reducing the secretion of M1-associated cytokines such as TNF-α and IL-6, while also activating STAT6/STAT3 signaling cascades to upregulate M2-associated markers (Yang et al., 2017). Importantly, the energy metabolism of M2 macrophages relies primarily on OXPHOS. Enhanced OXPHOS is accompanied by increased NAD^+^/NADH ratio and ATP levels. Changes in the intracellular NAD^+^/NADH ratio significantly affect the activity of SIRT1; elevated NAD^+^ enhances SIRT1 activity, which in turn regulates cellular metabolism and stress responses (Cerutti et al., 2014). SIRT1 can deacetylate and activate PGC-1α, contributing to the clearance of reactive oxygen species (ROS) induced by oxidative stress and alleviating oxidative damage (Wu QJ. et al., 2022). Paradoxically, recent studies indicate that PGC-1α may also suppress the IL-6/JAK2/STAT3 pathway, thereby limiting M2 polarization and attenuating fibrosis following hepatic ischemia-reperfusion injury (Zhang et al., 2023a).

#### 2.3.3 AMPK/mTORC1 axis: balancing M1-M2 metabolic transition

The AMP-activated protein kinase (AMPK) and mammalian target of rapamycin complex 1 (mTORC1) form a key metabolic-immunological regulatory axis, dynamically counterbalancing each other to coordinate macrophage polarization (Cheng et al., 2023). Specifically, during M1 polarization, mTORC1 promotes the transcription of glycolysis-related genes by activating hypoxia-inducible factor-1 alpha (HIF-1α), thereby driving the expression of pro-inflammatory cytokines (Wu et al., 2021).Conversely, AMPK inhibits mTORC1 activity via phosphorylation, reducing HIF-1α stability and disrupting glycolysis-dependent metabolism, ultimately suppressing M1 polarization and controlling inflammation (Cheng et al., 2023). Notably, low-molecular-weight fucoidan (LMWF), a natural product with anti-MRSA activity, has also been shown to induce macrophage polarization toward the M2 phenotype by activating the AMPK signaling pathway, significantly reducing the levels of inflammatory mediators such as IL-6 and TNF-α (Chen M. et al., 2025).Mechanistic studies indicate that the AMPK/mTORC1 axis regulates mitochondrial metabolic homeostasis to bidirectionally modulate macrophage polarization: AMPK activation favors the M2 reparative phenotype, whereas mTORC1 signaling promotes the M1 pro-inflammatory state.

#### 2.3.4 JAK-STAT pathway: cytokine-directed metabolic regulation

The Janus kinase (JAK)-signal transducer and activator of transcription (STAT) pathway serves as a central signaling hub for cellular responses to cytokines such as IL-6, IFN-γ, and IL-4. By integrating extracellular signals with nuclear gene expression programs, this pathway precisely regulates energy metabolism, immune responses, and tissue homeostasis (Xin et al., 2020). In psoriasis research, the research by Gao et al. found that ergothioneine (EGT) modulates macrophage polarization and ameliorates psoriasis by regulating the NF-κB/JAK-STAT3 signaling pathway: EGT downregulates the M1 marker CD86 and upregulates the M2 marker CD206 (Li A. et al., 2025).Metabolomic studies reveal that fructose, a typical ketohexose, suppresses STAT1 activation and blocks M1 macrophage polarization by reducing cytoplasmic and mitochondrial Ca^2+^ levels through a non-canonical metabolic pathway (Yan et al., 2024). IL-4, a key immunomodulatory cytokine, plays a crucial role in macrophage phenotype switching. Guo et al. found that IL-4 activates the JAK1/STAT6 phosphorylation cascade, downregulates pro-inflammatory cytokine expression, and significantly enhances M2 macrophage polarization (Guo et al., 2024). Under hypoxic conditions, metabolic reprogramming leads to succinate accumulation, which stabilizes HIF-1α and enhances STAT3 activity, thereby promoting anti-inflammatory cytokine expression. Targeted inhibition of the JAK1/STAT3/HIF-1α signaling axis effectively regulates energy metabolism, reduces pro-inflammatory mediator release, and ultimately mitigates bone and joint destruction (Zhang F. et al., 2024).

Notably, the JAK-STAT pathway exhibits pleiotropic regulatory characteristics in macrophage polarization. Previous studies on succinate dehydrogenase (SDH) have primarily focused on its role in promoting M1 polarization through ROS production. However, He et al. found that inhibition of SDH activity using dimethyl malonate (DMM) significantly upregulated the expression of M2 marker genes, including arginase-1 (Arg1) and chitinase-like protein 3 (Ym1), while simultaneously decreasing IL-1β levels and increasing IL-10 levels. This regulatory effect was mediated by STAT6 activation, ultimately promoting tissue repair (He et al., 2025) (Figure 1).

#### 2.3.5 Nrf2 pathway: antioxidant defense and M2 support

Nuclear factor erythroid 2-related factor 2 (Nrf2) serves as a key regulatory hub in oxidative stress defense, playing a critical role in balancing pro-inflammatory cytokine expression and reactive oxygen species (ROS) metabolism (Luchkova et al., 2024). Li et al. demonstrated that Nrf2 effectively suppresses M1 macrophage polarization and maintains redox homeostasis through a triple regulatory mechanism: reducing ROS levels, inhibiting IL-6/IL-1β transcriptional activity, and blocking the NF-κB activation pathway (Li Y. et al., 2025). Under pathological conditions, excessive ROS production and mitochondrial damage create a vicious cycle that exacerbates inflammatory cascades. Studies have shown that the mitochondria-targeted antioxidant mitoquinone (MitoQ) significantly reduces TNF-α and IL-6 levels by co-activating the Nrf2 signaling pathway and improving mitochondrial autophagy defects and dysfunction via the Nrf2/PINK1 axis (Cen et al., 2021). At the molecular level, Nrf2 directly regulates the expression of anti-inflammatory genes in M2 macrophages, and its specific activation has been confirmed to drive macrophages toward an M2 phenotype (Sha et al., 2023). Notably, Nrf2 knockout models revealed that the loss of this regulatory factor promotes M1 macrophage polarization through abnormal activation of the NF-κB/PPARγ signaling axis and disruption of autophagic flux (Luo et al., 2022). In the osteomyelitis microenvironment, Nrf2 exhibits dual regulatory functions: on one hand, it promotes M2 macrophage polarization to exert anti-inflammatory effects; on the other, it enhances ROS-scavenging enzyme activity to counteract RANKL-induced oxidative stress, thereby inhibiting osteoclast differentiation and facilitating bone repair. When Nrf2 function is impaired, excessive ROS accumulation activates the RANKL signaling pathway, accelerating osteoclastogenesis and bone resorption (Feng X. et al., 2023). Pathological cascade studies indicate that excessive oxidative stress directly leads to bone loss and structural damage. A recent study by Huang et al. (Huang L. et al., 2024)found that the natural compound picein not only improves the immune microenvironment by promoting M2 macrophage polarization but also enhances the osteogenic differentiation capacity of bone marrow mesenchymal stem cells (BMSCs) via the Nrf2/HO-1/GPX4 axis, significantly improving bone defect repair efficiency (Figure 1).

### 2.4 Mitochondrial metabolic dysfunction and its association with osteomyelitis pathology

#### 2.4.1 Mitophagy

During the progression of osteomyelitis, pathogen infection, oxidative stress, and chronic inflammation collectively disrupt mitophagy homeostasis, exacerbating immune-metabolic dysregulation and bone tissue destruction. As a critical quality control mechanism for selectively eliminating damaged mitochondria, mitophagy plays a pivotal role in maintaining mitochondrial network integrity, thereby modulating cellular metabolism and immune responses (Fu et al., 2020). Molecular studies have identified the PINK1/PARKIN signaling pathway as a central mediator of mitophagy. In osteomyelitis, *S. aureus* suppresses HDAC11 to upregulate IL-10 expression, thereby activating PINK1/Parkin-dependent mitophagy and clearing mitochondrial ROS to sustain its intracellular survival. This mechanism impairs macrophage bactericidal function, leading to persistent infection (Liu et al., 2022; Yang et al., 2025).Notably, macrophages serve as primary host cells for *S. aureus*, and their mitophagy levels directly influence reactive oxygen species (ROS)-mediated bactericidal activity, which is crucial for pathogen control (Li K. et al., 2023; Nan et al., 2024). Specifically, mitophagy mitigates mitochondrial ROS (mtROS) accumulation by removing damaged mitochondria, thereby impairing host antimicrobial efficacy (Li K. et al., 2023). Mechanistic studies have demonstrated that *S.aureus* infection significantly upregulates the expression of the anti-inflammatory cytokine interleukin-10 (IL-10), which, by promoting mitophagy and mtROS clearance, markedly enhances intracellular pathogen survival (Yang et al., 2025). Osteoclast resorption assays combined with tartrate-resistant acid phosphatase (TRAP) staining experiments revealed further suggests that modulating the AMPK/BNIP3/PINK1/PARKIN signaling axis to enhance mitophagy and reduce ROS accumulation effectively suppresses macrophage M1 polarization and osteoclast differentiation, ultimately alleviating bone loss (Wang W. et al., 2024). Therefore, in osteomyelitis, the primary pathogen *S. aureus* can enhance mitophagy to weaken macrophage bactericidal and anti-inflammatory functions, enabling immune evasion, escaping macrophage-mediated clearance, prolonging the course of infection, and contributing to chronic, refractory osteomyelitis.

#### 2.4.2 The bidirectional regulatory network of ROS

During infection, cellular antioxidant homeostasis is disrupted, leading to the abnormal accumulation of reactive oxygen species (ROS). As key byproducts of mitochondrial metabolism, ROS exert a dual regulatory effect on macrophage polarization: physiological levels promote pro-inflammatory M1 macrophage polarization, whereas excessive ROS induce cellular damage and functional impairments (Dong et al., 2022; Gu et al., 2017; Qing et al., 2020). In osteomyelitis pathogenesis, mitochondrial fission abnormalities and dysfunction collectively contribute to ROS accumulation, ultimately triggering infection-induced osteocyte death (Mendelsohn et al., 2023). Recent studies have uncovered that hyperactivation of the EGFR–MEK1/2 cascade downregulates mitochondrial ROS (mtROS) in macrophages by suppressing Chek2 expression, thereby impairing their bactericidal capacity; conversely, inhibition of this pathway markedly enhances *S.aureus* clearance and improves bone microarchitecture *in vivo* (Jin et al., 2024). It has also been shown that *S.aureus* suppresses HDAC11 to boost IL-10 production, which in turn promotes mitophagy and mtROS clearance, facilitating bacterial survival within macrophages (Yang et al., 2025). Another investigation demonstrated that PD-1/PD-L1 signaling activates mitophagy to reduce mtROS levels, thereby inhibiting macrophage antimicrobial function. Treatment with PD-1/PD-L1 neutralizing antibodies significantly decreases mitophagy in bone marrow macrophages, enhances bacterial eradication in bone tissue and implants, and reduces bone destruction in mice (Li K. et al., 2023).Precise modulation of ROS production has been shown to effectively control macrophage inflammatory responses and NLRP3 activation, providing a novel strategy for macrophage phenotype reprogramming (Wu X. et al., 2025). Collectively, these findings underscore the central role of ROS homeostasis in macrophage polarization regulation.

## 3 The pathological role of the mitochondrial metabolism–macrophage polarization axis in osteomyelitis

### 3.1 Dynamic changes in mitochondrial dynamics and macrophage polarization during osteomyelitis progression

**FIGURE 2:**
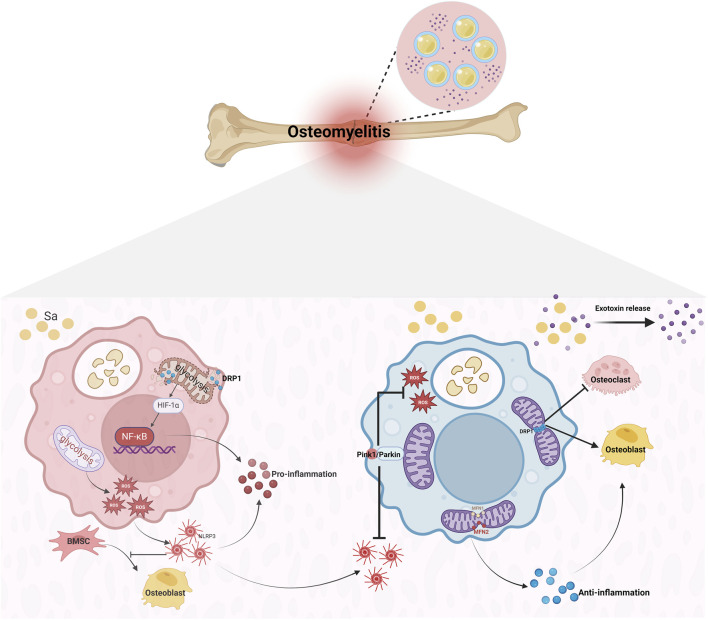
Macrophage Polarization and Pathological Progression in *S.aureus*-Induced Osteomyelitis. Upon *Staphylococcus aureus* invasion, macrophages engulf the pathogen, triggering Drp1-mediated mitochondrial fission and driving their polarization toward the pro-inflammatory M1 phenotype. As the infection progresses, upregulation of the fusion proteins Mfn1/2 shifts mitochondrial morphology from fragmented to fused, facilitating a transition to the reparative M2 phenotype. This M2 polarization promotes osteogenesis, dampens inflammation, and guides the lesion toward healing. However, biofilm formation encapsulates *Staphylococcus aureus*, enabling persistent bacterial survival and continuous toxin release. The result is necrotic bone, renewed mitochondrial fission, and heightened osteoclast activity. With bone resorption and formation occurring simultaneously, the lesion ultimately advances to chronic osteomyelitis.

During osteomyelitis progression, macrophages exhibit distinct immune functions through M1/M2 phenotypic switching. In the acute infection phase, pathogen invasion triggers rapid mitochondrial fission—mediated primarily by Drp1—resulting in a fragmented mitochondrial network (Susser et al., 2023). This fission event initiates an “immune defense program,” in which resting (M0) macrophages polarize to the M1 phenotype. M1 macrophages rapidly activate via the NF-κB pathway and upregulate HIF-1α signaling, driving the release of pro-inflammatory cytokines such as TNF-α and IL-1β to establish a multifaceted immune barrier. This process not only enhances the inflammatory response but also facilitates the recruitment of neutrophils, T cells, and other immune cells, forming a pathogen clearance network (Xu et al., 2022). However, excessive M1 polarization amplifies inflammatory cascades, with mitochondrial ROS release activating the NLRP3 inflammasome in a positive feedback loop. This not only suppresses the osteogenic differentiation capacity of bone marrow mesenchymal stem cells (BMSCs) but also exacerbates structural damage to bone tissue through sustained inflammation (Zhao et al., 2024). Molecular studies have shown that activating mitophagy via the PINK1/PARKIN pathway effectively inhibits NLRP3 activation, thereby promoting macrophage M2 polarization and alleviating inflammation (Liu et al., 2025). During the chronic inflammatory phase, macrophage mitochondria transition from a fragmented to a fused state, characterized by mitochondrial elongation and network formation driven by upregulation of fusion proteins Mfn1 and Mfn2. This morphological shift supports M2 polarization, which is dominated by FAO and OXPHOS (Kawano et al., 2023), and promotes inflammation resolution and bone regeneration through the secretion of anti-inflammatory cytokines such as IL-10 and other tissue repair–associated factors (Chen et al., 2023). Notably, *S.aureus*–induced osteomyelitis exhibits a unique pathology: the acute inflammatory response fails to fully clear the pathogen, and the comparatively weaker phagocytic capacity of M2 macrophages allows biofilm formation and sustained toxin release, ultimately leading to chronic, recalcitrant infection foci (Masters et al., 2022; Muthukrishnan et al., 2019) (Figure 2) (Table 2)

**TABLE 2:** Comparison of metabolic remodeling in acute and chronic osteomyelitis.

Characteristic	Acute osteomyelitis	Chronic osteomyelitis
Metabolic Pathway	Predominantly glycolysis, reduced oxidative phosphorylation	Primarily oxidative phosphorylation, partial restoration
Mitochondrial Function	High ROS production, increased mitochondrial fission	Decreased mitochondrial bioenergetics, mitochondrial damage
Macrophage Polarization	M1-dominated, pronounced pro-inflammatory features	M2-dominated, limited reparative capacity
Signaling Regulation	HIF-1α and mTOR activation drive pro-inflammatory metabolism	AMPK and PGC-1α mediate metabolic adaptation
Inflammatory Microenvironment	Highly inflammatory, prioritizing pathogen clearance	Low-grade inflammation, coexistence of fibrosis and residual pathogens
Tissue Repair	Destructive inflammation leads to bone loss	Impaired repair, resulting in fibrosis and failed bone remodeling

### 3.2 Mitochondrial dynamics imbalance drives inflammation and bone destruction

#### 3.2.1 Metabolic crosstalk between macrophages and osteoclasts

In osteomyelitis, the metabolic interplay between macrophages and osteoclasts is a dynamic process driven by metabolic reprogramming, inflammatory signaling, and microenvironmental cues. Early in infection, M1 macrophages rely on glycolysis to rapidly produce ATP and secrete pro-inflammatory cytokines that positively regulate osteoclast differentiation and bone resorption. However, prolonged inflammation induces osteoclast apoptosis via upregulation of pro-apoptotic genes such as *Bax* and *Bak* (Wu CS. et al., 2025). During *S.aureus* infection, macrophages suppress HDAC11 to upregulate IL-10, which triggers PINK1/Parkin-dependent mitophagy, clears mtROS, and enables bacterial immune evasion—thereby indirectly disrupting bone remodeling (Yang et al., 2025). Inflammatory cytokines, notably IL-1β, released by macrophages further promote osteoclast differentiation and maturation (Wu YL. et al., 2022). Cyclooxygenase-2 (COX-2) is also upregulated in osteomyelitis; its inhibitor celecoxib mitigates bone loss by modulating immune cell populations (e.g., reducing MDSCs) and inhibiting osteoclastogenesis (Chen Y. et al., 2025).

Impaired bone healing in diabetic models has been traced to aberrant macrophage–BMSC communication: macrophage-derived exosomal miR-144-5p targets *Smad1* to inhibit osteogenic differentiation, whereas blockade of this miRNA reverses the repair defect (Zhang et al., 2021). Excessive activation of the EGFR–MEK1/2 cascade by *S.aureus* lowers mtROS in macrophages, impairing their bactericidal function; MEK1/2 inhibition in murine osteomyelitis reduces bacterial load and alleviates bone destruction (Jin et al., 2024).

Finally, mitochondrial complex I dysfunction, modeled by *Ndufs4* deletion, shifts metabolism from FAO to glycolysis. This enhances macrophage activation and inflammation while impairing osteoclast formation and bone resorption, highlighting the importance of intact mitochondrial respiration for balanced bone remodeling (Jin et al., 2014).

#### 3.2.2 Mitochondrial dynamics modulating the metabolic axis

Mitochondrial fission refers to the structural separation of damaged mitochondria from healthy ones, whereas mitochondrial fusion involves the reorganization of membrane structures and mixing of contents between depolarized mitochondria (Liu J. et al., 2024). This dynamic process is regulated by key proteins including mitofusin 1/2 (MFN1/2) and OPA1 for fusion, and dynamin-related protein 1 (DRP1, encoded by DNM1L) and fission protein 1 (FIS1) for fission. DRP1, a GTPase, is the principal executor of mitochondrial fission. When the fission–fusion balance is disrupted, mitochondrial quality control collapses, leading to dysfunction, cell death, and tissue damage (Liu J. et al., 2024).

In osteomyelitis models, imbalanced fission and fusion result in abnormally elevated mtROS in macrophages, significantly inhibiting osteoclast differentiation (Jin et al., 2014). Mechanistically, DRP1 overexpression causes mitochondrial membrane potential collapse and structural fragmentation, impairing osteoblast function; conversely, DRP1 inhibition effectively alleviates oxidative stress–induced osteogenic defects (Zhang et al., 2017). Notably, DRP1 promotes osteoclast differentiation via the c-Fos–NFATc1 signaling axis, and its suppression blocks LPS-induced osteoclastogenesis (Jeong et al., 2021). MFN2-mediated remodeling of endoplasmic reticulum–mitochondria contacts regulates mitochondrial Ca^2+^ uptake and induces cytosolic Ca^2+^ oscillations that further drive osteoclastogenesis (Ballard et al., 2020).Clinical studies have shown that the extent of mitochondrial damage in osteoblasts from osteomyelitis patients correlates with an increased RANKL/OPG ratio, which directly determines the balance between bone resorption and formation (Granata et al., 2022; Ferver et al., 2021). Moreover, resveratrol enhances macrophage mitophagy via the SIRT1–PGC-1α pathway, upregulates MFN2 expression to promote mitochondrial fusion (Wu SK. et al., 2024), and boosts PINK1/Parkin-mediated mitophagy to accelerate damaged mitochondrial clearance and reduce mtROS accumulation, thereby driving macrophages toward an anti-inflammatory M2 phenotype. These effects optimize mitochondrial dynamics and energy metabolism in the osteomyelitis microenvironment, ultimately influencing osteoclast differentiation (Jin et al., 2014). Recent work has also identified the Sigma-1 receptor at mitochondria-associated membranes (MAM) as a negative regulator of osteoclastogenesis, suggesting that targeting ER–mitochondria interactions may offer novel therapeutic avenues for osteomyelitis (Ballard et al., 2020).

### 3.3 Interaction between pathogen evasion and aberrant Macrophage polarization

Many pathogens employ diverse mechanisms to suppress M1 macrophage polarization, thereby weakening host immune defenses. For example, mycobacteria can inhibit the IFN-γ signaling pathway, maintaining macrophages in an M2-like state that favors intracellular survival. This inhibition is thought to occur via intracellular signaling modulation—such as suppressing STAT1 activation—to reduce the expression of M1 markers. Certain pathogens actively induce M2 polarization to create an immune microenvironment conducive to their survival and evasion. For instance, the mannose-capped lipoarabinomannan (ManLAM) of *Mycobacterium tuberculosis* stimulates TGF-β production, driving macrophage conversion from M1 to M2. This shift benefits the pathogen by exploiting the relatively weak bactericidal capacity and anti-inflammatory, tissue-repairing metabolism of M2 macrophages (Ren et al., 2017).In chronic osteomyelitis, pathogens can persist within overly polarized M2 macrophages, escaping immune clearance and antibiotic treatment. Emerging therapies therefore focus on macrophage phenotype reprogramming—shifting M2 back to M1 to enhance bactericidal activity (Tian et al., 2023). Mechanistic studies show that inhibiting MAP3K1 and NF-κB signaling can promote M1-to-M2 repolarization, thereby reducing inflammation in osteomyelitis (Dai et al., 2024). Additionally, SETD2 regulates HIF-1α expression to modulate glycolysis in osteomyelitis-associated macrophages, influencing their function and polarization (Zhu D. et al., 2024).As key players in innate immunity, macrophages both clear pathogens and orchestrate inflammation resolution and tissue repair. Macrophage-deficient models exhibit markedly reduced bone density, underscoring their central role in bone homeostasis (Kang et al., 2020). Under physiological conditions, M2 macrophages support bone formation by promoting MSC-to-osteoblast differentiation (Mi et al., 2022), whereas M1 macrophages serve as osteoclast precursors in bone resorption (Hu et al., 2023). In osteomyelitis, an imbalance in M1/M2 polarization disrupts the equilibrium between osteogenesis and osteoclastogenesis, establishing a vicious “inflammation–bone destruction” cycle. Therefore, precisely modulating macrophage polarization homeostasis represents a critical therapeutic target for improving clinical outcomes.

## 4 Targeting mitochondrial metabolism for osteomyelitis treatment

### 4.1 Metabolic interventions for regulating macrophage polarization

**FIGURE 3:**
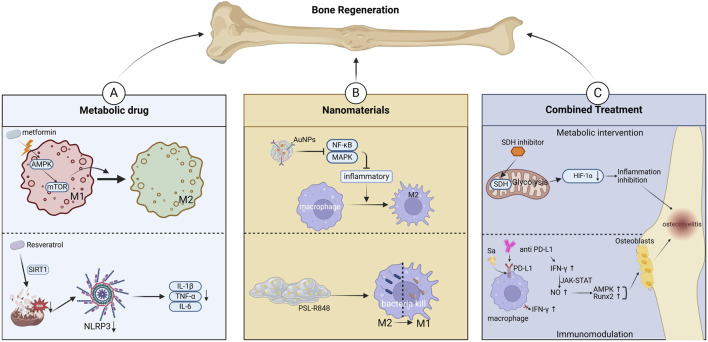
Targeting mitochondrial metabolism for osteomyelitis Treatment. **(A)** Metabolic interventions for regulating Macrophage Polarization. **(B)** Nanomaterials and drug delivery Systems. **(C)** Combination therapy Strategies.

Reactive oxygen species (ROS) play a central role in innate immune responses and inflammation. Elevated mitochondrial ROS levels lead to loss of mitochondrial membrane potential (MMP), triggering mitochondrial dysfunction and reduced cell viability (Soliman et al., 2024). Moreover, the combined effect of increased ROS and decreased MMP significantly decreases cell survival and induces apoptosis (Chen et al., 2022b).In osteomyelitis, excessive ROS production exacerbates inflammation through mitochondrial damage mechanisms (Andrieux et al., 2021). Chronic osteomyelitis patients often exhibit mitochondrial pathology, including swelling, cristae loss, and decreased matrix density. These alterations not only impair macrophage phagocytic and bactericidal functions but also promote inflammation by releasing damage-associated molecular patterns (DAMPs) (Mendelsohn et al., 2023). CRISPR/Cas9‐mediated gene knockout studies and metabolic flux analyses have shown that N-acetyl-L-cysteine (NAC) exerts tissue‐protective effects by scavenging cytosolic and mitochondrial ROS, preventing macrophage cytoplasmic swelling and membrane rupture, and thereby reducing the expression of pyroptosis‐associated proteins and pro‐inflammatory cytokines while upregulating anti‐inflammatory mediators (Zhang et al., 2024b; Zhang D. et al., 2020). Mitochondrial metabolic dysfunction influences immune cell function through multiple pathways. AMP-activated protein kinase (AMPK), a key regulator of mitochondrial energy homeostasis, has been shown to suppress NLRP3 inflammasome activation, restore mitochondrial function, and reduce oxidative stress (Yang et al., 2023). Additionally, AMPK activation induces metabolic reprogramming, enhancing mitochondrial OXPHOS to inhibit inflammation progression (Jin et al., 2020). In acute suppurative osteomyelitis, staphylococcal protein A alleviates disease progression by inhibiting SETD2-mediated upregulation of glycolysis, thereby suppressing hBMSC osteogenic differentiation and M1 macrophage polarization (Zhu D. et al., 2024). During the subacute phase of *S.aureus* osteomyelitis, PD-1/PD-L1 signaling enhances mitophagy, reduces mtROS levels, and impairs macrophage bactericidal activity; PD-1/PD-L1 blockade restores macrophage polarization and bacterial clearance in osteomyelitis (Li K. et al., 2023).Notably, mitochondrial fission is significantly increased during osteomyelitis. The resulting mitochondrial fragments promote autophagy, alleviating oxidative stress-induced mitochondrial dysfunction and preserving macrophage function (He et al., 2019). Resveratrol has been found to enhance mitophagy, reduce ROS levels, and inhibit NLRP3 inflammasome activation, consequently decreasing pro-inflammatory cytokine expression and alleviating inflammation (Liu et al., 2025).Similarly, activation of the Nrf2 signaling pathway has been shown to maintain mitochondrial homeostasis in alveolar macrophages by promoting mitophagy, thereby preventing NLRP3 inflammasome activation and limiting inflammation (Dong et al., 2021). Moreover, succinate dehydrogenase (SDH), a key enzyme in mitochondrial metabolism, plays a crucial role in the electron transport chain. SDH deficiency in macrophages reduces hypoxia-inducible factor-1α (HIF-1α) stability, leading to sustained IL-1β expression and prolonged inflammation (Gobelli et al., 2023; Fuhrmann et al., 2019). Inhibiting mitochondrial SDH activity disrupts HIF-1α-mediated glycolysis, deactivates the NLRP3 inflammasome, and attenuates inflammation (Zhu XX. et al., 2024). Not only have numerous *in vitro* and animal studies elucidated the roles of mitochondrial fission–fusion dynamics, ROS generation, and metabolite signaling in macrophage polarization, but clinical observations also provide compelling evidence. For example, septic patients exhibit significantly elevated levels of circulating cell-free mtDNA in plasma, which correlate positively with SOFA scores and mortality (Scozzi et al., 2021), suggesting its potential diagnostic and prognostic value during systemic inflammation complicating osteomyelitis. Furthermore, electron microscopy and immunohistochemical analyses of bone tissue from patients with chronic suppurative osteoarticular infections revealed increased Drp1 expression and pronounced mitochondrial fragmentation in infiltrating macrophages, underscoring the clinical relevance of mitochondrial dynamics regulation (Mendelsohn et al., 2023).These findings suggest that targeting mitochondrial metabolism is an emerging therapeutic strategy for osteomyelitis (Figure 3).

### 4.2 Nanomaterials and drug delivery systems

The dynamic changes in macrophage polarization play distinct regulatory roles at different pathological stages of osteomyelitis. Within the infectious microenvironment, macrophage phenotype transition, characterized by the secretion of pro-inflammatory factors such as macrophage inflammatory protein-1α (MIP-1α) and the promotion of osteoclastogenesis, exacerbates inflammation and bone destruction (Trouillet-Assant et al., 2015; Dapunt et al., 2014). Recent studies have focused on developing various materials and drugs to modulate mitochondrial metabolism, thereby regulating macrophage polarization and improving clinical outcomes in osteomyelitis. Studies have demonstrated that drugs such as celecoxib suppress the NF-κB pathway, reducing M1 macrophage activity while promoting M2 polarization, ultimately alleviating inflammatory bone damage (Weber et al., 2021; Wang W. et al., 2022). Strontium ions (Sr^2+^) activate the PI3K/AKT/mTOR signaling pathway, enhancing mitochondrial function in macrophages and inducing their transition to the M2 phenotype, thereby creating an immune microenvironment conducive to tissue repair (Qiu et al., 2024). Gold nanoparticles exert a dual inhibitory effect on the NF-κB and MAPK signaling pathways, significantly driving macrophage polarization toward the anti-inflammatory M2 phenotype and effectively counteracting LPS-induced inflammation. This immunomodulatory action further promotes the osteogenic differentiation of bone marrow mesenchymal stem cells (BMSCs) *in vitro*. Mechanistically, gold nanoparticles specifically upregulate the expression of the macrophage surface receptor TREM2, enhancing phagocytic activity against *S.aureus*. Concurrently, TREM2 signaling accelerates autophagosome–lysosome fusion, facilitating intracellular pathogen degradation and establishing an antibacterial “recognition–phagocytosis–clearance” loop. In models of osteomyelitis and infected skin wounds, gold nanoparticles achieve dual therapeutic benefits by both controlling infection—via modulation of the inflammatory microenvironment—and activating bone regeneration pathways (Peilin et al., 2023; Fu et al., 2021). In recent years, advancements in nanomaterial design have further expanded therapeutic strategies. Biomimetic piezoelectric nanocomposites leverage both reactive oxygen species (ROS)-mediated antibacterial effects and piezoelectric properties to reprogram macrophages toward the M2 phenotype while activating calcium ion channels to promote mesenchymal stem cell osteogenic differentiation, significantly accelerating bone regeneration (Roy et al., 2025). Additionally, an emodin-based multifunctional nanoplatform facilitates ROS-dependent bacterial membrane disruption and regulates M1 polarization via the cAMP/cGMP-PKG pathway. After pathogen clearance, it shifts toward M2 polarization, fostering osteogenic repair (Li Z. et al., 2025). For drug-resistant bacterial infections, metal ion-antibiotic co-loaded nanoplatforms effectively disrupt methicillin-resistant *S.aureus* (MRSA) membrane structures, inhibit glycolysis, and interfere with energy metabolism through multiple mechanisms, significantly reducing both intracellular and extracellular bacterial burden while restoring antibiotic sensitivity (Lv et al., 2025). Beyond nanomaterials, exosome-based therapies have emerged as a promising approach to modulate macrophage M2 polarization in inflammatory lesions, thereby suppressing pro-inflammatory cytokine release and promoting tissue repair (Chen Y. et al., 2024). Recent studies have also highlighted that specifically functionalized hydrogels can reverse macrophage polarization from M2 to M1, enabling intracellular bacterial clearance, thus offering a novel strategy for osteomyelitis treatment. These innovative delivery systems hold significant potential for immune microenvironment modulation and drug-resistant pathogen eradication (Figure 3).

### 4.3 Combination therapy strategies

Macrophage polarization is a central regulator of the immune microenvironment in osteomyelitis, and its metabolic reprogramming has emerged as a key target for combination therapy. Studies have confirmed that succinate dehydrogenase (SDH) and its metabolic product, succinate, play a crucial regulatory role in macrophage phenotype transitions. SDH inhibitors effectively suppress the glycolytic pathway and HIF-1α expression through a dual mechanism, leading to the inactivation of the NLRP3 inflammasome. This process attenuates M1 macrophage-mediated inflammation while promoting M2 polarization (Sun HJ. et al., 2024). During persistent infections, pathogens may evade immune surveillance by inducing programmed death-ligand 1 (PD-L1) expression. The use of PD-L1/PD-1 inhibitors, such as PD-L1 monoclonal antibodies, can block this immunosuppressive signal, restore interferon-γ (IFN-γ) levels, and enhance macrophage bactericidal activity (Wang et al., 2024b). IFN-γ further activates the JAK-STAT signaling pathway to promote nitric oxide synthesis, stimulating the p38 MAPK and Runx2 signaling cascades, which ultimately enhance osteogenic differentiation (Wang H. et al., 2023).The development of advanced delivery systems provides technological support for combination therapies. In a post-traumatic osteomyelitis (PTO) model, Wenting Zhang’s team developed a ROS-responsive hydrogel co-loaded with quorum-sensing (QS) inhibitors and antimicrobial peptides, which effectively eradicated nearly all bacteria while significantly promoting bone regeneration (Zhang W. et al., 2024).Such “hydrogel + antibiotics + metabolic regulators” triple-combination strategies exhibit great potential for clinical translation. Future research may focus on developing similar nanomaterials or hydrogel-based targeted therapies. Building on the bone regeneration platform developed by Xu Zhengjiang’s team, an ordered 5–20 nm mesoporous bioactive glass (MBG) shell was deposited onto hydroxyapatite (HAp) nanoparticles via sol–gel and self-assembly techniques. This design increased vancomycin loading over threefold compared to conventional 58S glass and yielded a sustained, controlled release profile. Si^4+^ ions released from the MBG shell not only mitigate drug toxicity to osteoblasts but also upregulate osteogenic genes (e.g., ALP, Runx2), enhancing cell proliferation and differentiation. The HAp core provides a biomimetic osteoconductive scaffold that further promotes cell adhesion and mineralization. In a chronic osteomyelitis rat model, this carrier achieved a synergistic “anti-infection and pro-regeneration” effect, combining potent antibacterial activity with accelerated bone defect repair (Xu et al., 2021). Localized delivery platforms, including degradable hydrogels, MBG bone cement, and nanomaterials co-loaded with antibiotics or metabolic regulators, hold significant promise for the future treatment of osteomyelitis (Figure 3) (Table 3).

**TABLE 3:** Summary Table of potential therapeutic targets for mitochondrial metabolism regulation.

Target/Mechanism	Function	Drugs/Regulatory strategies	Potential clinical prospects	References
AMPK	Activates mitochondrial energy metabolism, inhibits oxidative stress, promotes oxidative phosphorylation	metformin	Inflammation control in osteomyelitis	(Yang et al., 2023; Jin et al., 2020)
mTOR	Regulates autophagy and protein synthesis, influences macrophage polarization	Rapamycin, Sr^2+^	Immune dysregulation and repair in osteomyelitis	(Fan et al., 2022; Zhao et al., 2023; Qiu et al., 2024)
HIF-1α	Promotes glycolytic metabolism, regulates pro-inflammatory responses in M1 macrophages	HIF-1α inhibitors (e.g., YC-1)	Inflammatory response control in osteomyelitis	(Van den Bossche et al., 2016; Zhu et al., 2024a)
PGC-1α	Enhances mitochondrial biogenesis, promotes anti-inflammatory properties of M2 macrophages	PGC-1α activator (e.g., Ckip-1)	Repair in osteomyelitis	(Feng et al., 2023a; Huang et al., 2024b)
SDH	Regulates mitochondrial oxidative phosphorylation	SDH inhibitors (e.g., DMM)	Inflammation control in osteomyelitis	(Gobelli et al., 2023; Fuhrmann et al., 2019; Zhu et al., 2024b)
Mitophagy	Clears damaged mitochondria, reduces ROS production	Parkin activators, Nrf2 activators	Inflammation control in osteomyelitis	(He et al., 2019; Dong et al., 2021; Song et al., 2023)
ROS	Promotes oxidative stress and pro-inflammatory cytokine expression	NAC	Inflammation control in osteomyelitis	(Zhang et al., 2024b; Zhang et al., 2020a)
FAO	Provides energy for M2 macrophage polarization	CPT1A activator	Inflammation control and reparative effects in osteomyelitis	(Li et al., 2024a; Soliman et al., 2020)
MMP	Maintains mitochondrial function, regulates macrophage polarization, supports oxidative phosphorylation	JC-1-labeled drugs	Macrophage function regulation in osteomyelitis	(Chen et al., 2022b)
TCA Cycle	Modulates mitochondrial metabolic flux, influences macrophage polarization balance	TCA cycle-related metabolic regulators	regulation of inflammation and metabolic disorders in osteomyelitis	Liu and Ho (2018)

## 5 Potential applications of mitochondrial metabolic regulation in other orthopedic diseases

### 5.1 Inflammatory joint diseases: osteoarthritis and rheumatoid arthritis

Osteoarthritis (OA), the most prevalent degenerative joint disease, is a major cause of joint dysfunction and chronic pain in adults (Chen et al., 2017). Its pathogenesis involves the interplay between inflammatory cascades and metabolic homeostasis imbalance. Studies have demonstrated that mitochondrial metabolism directly influences inflammation and cartilage degradation in OA by regulating macrophage polarization between the M1 and M2 phenotypes (Qing et al., 2020). At the molecular level, M1 macrophages exhibit a glycolysis-dominant metabolic profile, characterized by reactive oxygen species (ROS) bursts and succinate accumulation. This process leads to the secretion of pro-inflammatory cytokines such as IL-1β and TNF-α, which activate matrix metalloproteinases, ultimately driving cartilage matrix degradation and chondrocyte apoptosis. Conversely, M2 macrophages primarily rely on FAO and OXPHOS to exert anti-inflammatory and tissue-repair functions through the release of IL-10 and TGF-β(131). In OA, mitochondrial metabolic dysregulation exacerbates inflammation. Excessive ROS production not only directly damages chondrocytes but also activates the NLRP3 inflammasome, reinforcing M1 polarization and forming a positive feedback loop of synovial inflammation (Zhang Z. et al., 2023).Persistent inflammation-induced glycolytic reprogramming and mitochondrial dysfunction contribute to immune dysregulation and impaired bone regeneration. Notably, defective mitophagy leads to the accumulation of damaged mitochondria, further amplifying oxidative stress and metabolic imbalance (Li X. et al., 2023). Targeting mitochondrial metabolism offers a promising therapeutic strategy for OA. Restoring macrophage polarization balance (inhibiting M1 and promoting M2 phenotypes) can effectively enhance cartilage repair and mitigate joint inflammation. Studies have demonstrated that songorine can induce metabolic reprogramming by suppressing glycolysis and enhancing OXPHOS, thereby shifting M1 macrophages towards an M2 phenotype, reducing cartilage damage, and alleviating synovitis (He et al., 2024). PGAM5, a serine/threonine phosphatase located on the mitochondrial membrane, has been found to promote M1 polarization via the AKT-mTOR/p38/ERK pathway while inhibiting M2 polarization through the STAT6-PPARγ pathway; its genetic knockout significantly improves OA pathology (Liu Y. et al., 2024). Furthermore, MAGL inhibitors restore mitophagy levels, facilitating the transition of M1 to M2 macrophages, effectively reducing synovial inflammation scores and pain thresholds in murine models (Gu et al., 2023). Additionally, SIRT3 deficiency has been shown to accelerate M1 polarization and cartilage degeneration, whereas its activation, along with honokiol treatment, significantly delays OA progression (Zhang et al., 2023c). Functionalized extracellular matrix (ECM) hydrogels restore mitochondrial morphology and markedly enhance the membrane potential of damaged mitochondria, thereby inhibiting mtROS accumulation and improving mitochondrial function to promote M2 polarization. This effect concurrently suppresses inflammatory cytokine expression and reduces cartilage matrix degradation (Chen Z. et al., 2024).Collectively, these studies highlight the dual role of mitochondrial metabolism in OA: it serves as both a key regulator of macrophage polarization balance and a central mediator of the inflammation–repair dynamic transition.

Rheumatoid arthritis (RA), a chronic autoimmune disorder characterized by persistent synovial inflammation and progressive joint destruction, is also closely associated with imbalances in macrophage polarization, where mitochondrial metabolism plays a critical regulatory role (Zheng et al., 2024). As described above, M1 macrophages exacerbate synovial inflammation and bone erosion by secreting pro-inflammatory cytokines that activate the NLRP3 inflammasome (Zhang Z. et al., 2023). In contrast, M2 macrophages secrete anti-inflammatory factors that facilitate inflammation resolution and tissue regeneration (Mills, 2015; Gobert and Wilson, 2012; Zhang and Ji, 2023).However, RA synovial macrophages exhibit characteristic mitochondrial dysfunction, including enhanced mitochondrial fission, excessive ROS production, and impaired autophagy, which collectively reinforce M1 polarization and sustain inflammatory cascades (Clayton et al., 2021; Cutolo et al., 2022). Metabolic reprogramming of macrophages has thus emerged as a promising therapeutic approach for RA. Experimental studies indicate that a low-protein diet can activate the NRF2/SIRT3/SOD2 signaling axis, effectively suppressing M1 polarization in synovial macrophages, reducing mitochondrial ROS levels, and alleviating synovial inflammation and joint damage in RA models (Fu et al., 2024). Furthermore, plant-derived bioactive compounds such as punicalagin have been shown to regulate macrophage polarization by downregulating M1 markers while upregulating M2 markers, demonstrating potent anti-arthritis effects *in vivo* (Ge et al., 2022).Traditional herbal extracts, including Gentiana lutea ethyl acetate extract and Ermiao San, have been reported to suppress NLRP3 inflammasome activation, thereby inhibiting M1 polarization and improving the inflammatory microenvironment in RA synovium (Liu et al., 2023; Zhang et al., 2024d).Notably, peroxisome proliferator-activated receptor gamma (PPARG) has been implicated in enhancing mitophagy-mediated ROS clearance, thus restoring macrophage polarization balance and representing a novel therapeutic target for RA (Geng et al., 2024).

Given the shared mitochondrial metabolic mechanisms underlying OA and RA, future therapeutic strategies could focus on the following directions: 1)Developing small-molecule compounds that target glycolysis/OXPHOS balance. 2)Utilizing metabolic intermediates to regulate TCA cycle activity and counteract M1/M2 polarization imbalance. 3)Constructing biomimetic nanocarriers or functionalized hydrogels to achieve targeted delivery of ROS scavengers and SIRT1/3 activators, thereby restoring mitochondrial function in infected bone tissue macrophages. 4)Combining autophagy activators with NLRP3 inflammasome inhibitors to collaboratively disrupt the inflammation–metabolism vicious cycle. Through these precise regulatory strategies, it may be possible to achieve controlled intervention of macrophage polarization, thereby optimizing therapeutic outcomes in inflammatory joint diseases. Despite their promise, these strategies face several critical challenges and limitations. First, developing small‐molecule modulators of glycolysis/OXPHOS balance (e.g., songorine analogs) must overcome poor delivery efficiency and targeting within bone tissue: its dense structure and unique microenvironment can impede drug penetration, while complex *in vivo* distribution, metabolism, and excretion may compromise efficacy (Zhang M. et al., 2024). Second, using metabolic intermediates (e.g., citrate, carnitine) to regulate the TCA cycle risks broad perturbation of cellular metabolism and may itself induce metabolic imbalance due to the complexity of *in vivo* homeostasis (Wen et al., 2025). Third, biomimetic nanocarriers and functionalized hydrogels must demonstrate biocompatibility, stability, and the ability to selectively target macrophages within infected bone. Moreover, combining autophagy activators (e.g., icariin) with NLRP3 inflammasome inhibitors (e.g., PPARγ agonists) might trigger unintended cellular responses, as their interactions and effects on non–macrophage cell types remain unclear and could lead to off‐target toxicity (Wen et al., 2025). The heterogeneity of bone‐resident macrophage subsets—each with distinct functions and metabolic profiles at different disease stages—may also render a given intervention effective for some subsets but ineffective or harmful for others (Zhang M. et al., 2024). Finally, the complex bone microenvironment, which includes osteoblasts, osteoclasts, chondrocytes, and multiple intercellular signaling networks, can modulate macrophage behavior and influence the outcome of metabolic interventions (Zhang M. et al., 2024). In summary, although grounded in sound scientific rationale and offering considerable potential, these approaches require further investigation and optimization to ensure their safety and efficacy in treating orthopedic diseases.

### 5.2 Disorders of bone metabolic imbalance: osteoporosis and intervertebral disc degeneration

Osteoporosis (OP) is a systemic bone metabolic disorder characterized by decreased bone mineral density and impaired bone microstructure, leading to increased pathological bone fragility. This condition is closely associated with immune dysregulation, particularly the imbalance of macrophage polarization, which plays a pivotal role in its pathogenesis (Muñoz et al., 2020). Studies have demonstrated that M1 macrophages exacerbate bone resorption through two primary mechanisms (Lew and Waldvogel, 2004): secreting pro-inflammatory cytokines such as TNF-α and IL-6 and (Kavanagh et al., 2018) differentiating into mature osteoclasts, thereby directly enhancing osteoclastic activity. Conversely, M2 macrophages promote osteogenesis by releasing osteogenic factors such as bone morphogenetic protein-2 (BMP-2) and insulin-like growth factor-1 (IGF-1), which facilitate the differentiation of mesenchymal stem cells (MSCs) into osteoblasts, ultimately enhancing bone mineralization (Hu et al., 2023; Muñoz et al., 2020). The central regulatory mechanism underlying this process involves mitochondrial metabolism, which finely modulates macrophage polarization and function. As previously described, excessive pro-inflammatory cytokines secreted by M1 macrophages significantly enhance osteoclast activity via RANKL signaling, accelerating bone loss (Liu and Ho, 2018; Van den Bossche et al., 2016; Vannella and Wynn, 2017). This metabolic–inflammatory cascade forms a vicious cycle: excess ROS not only directly damages bone cells but also amplifies inflammatory signaling through the NF-κB pathway. In contrast, M2 macrophages secrete anti-inflammatory mediators that promote osteogenic differentiation and bone matrix mineralization (Gobert and Wilson, 2012). OP patients typically exhibit an imbalance in the M1/M2 macrophage ratio, characterized by an abnormal increase in M1 macrophages and a reduction in M2 macrophages, accompanied by impaired mitophagy and the accumulation of damaged mitochondria. This metabolic disruption is intertwined with alterations in the bone marrow microenvironment, where an increased proportion of senescent bone marrow mesenchymal stem cells (BMSCs) and macrophages leads to a dominance of the pro-inflammatory M1 phenotype and a deficiency in the reparative M2 phenotype, establishing a state of chronic low-grade inflammation. Recent studies have highlighted potential therapeutic strategies targeting these mechanisms. Icariin (ICA) has been shown to reverse osteogenic dysfunction in senescent BMSCs by activating the autophagy pathway, significantly mitigating bone loss in osteoporotic mouse models (Bai et al., 2023). Astragaloside IV (AS-IV) exerts dual effects by inhibiting M1 polarization-associated mitochondrial dysfunction while simultaneously promoting M2 polarization and delaying macrophage senescence, thereby enhancing the osteogenic differentiation potential of BMSCs (Li M. et al., 2024). Additionally, key metabolic regulators offer promising therapeutic perspectives for OP treatment:1)Carnitine deficiency exacerbates oxidative stress and promotes excessive osteoclast activation, while its supplementation effectively suppresses M1 polarization and osteoclast differentiation (Yang T. et al., 2024).2) Citrate, by inhibiting key glycolytic enzymes, reprograms macrophage metabolism toward an M2-dominant OXPHOS phenotype, thereby maintaining bone metabolic homeostasis (Wu X. et al., 2024). Collectively, these findings suggest that targeting mitochondrial metabolism-driven macrophage polarization reprogramming may represent an innovative strategy to counteract OP pathogenesis.

Intervertebral disc degeneration (IDD) is a degenerative spinal disorder characterized by structural deterioration of the intervertebral disc, loss of function, and neuropathic pain. Its pathological progression is closely linked to chronic inflammation and macrophage polarization imbalance (Li et al., 2022; Koroth et al., 2023). Studies indicate that macrophage polarization exhibits a dual role in the IDD microenvironment: M1 macrophages aggravate ECM degradation and inhibit nucleus pulposus (NP) cell proliferation through the secretion of pro-inflammatory cytokines such as TNF-α and IL-1β, whereas M2 macrophages mitigate disc degeneration by releasing anti-inflammatory cytokines and growth factors (Li et al., 2022; Zhang H. et al., 2020). Recent research has uncovered mitochondrial metabolic reprogramming as a key mechanism linking macrophage polarization to IDD progression, influencing disease outcomes by modulating the energy metabolism networks of both intervertebral disc cells (NP and annulus fibrosus cells) and immune cells (Viola et al., 2019; Qing et al., 2020; Chen et al., 2024c). On a molecular level, mitochondrial dysfunction within the degenerative IDD microenvironment establishes a pathological feedback loop that perpetuates inflammation: 1)Mitochondrial dysfunction in intervertebral disc cells, particularly NP and annulus fibrosus cells, along with macrophages, directly affects inflammation, tissue repair, and the progression of disc degeneration (Koroth et al., 2023). 2)In degenerated NP cells, abnormally high expression of hypoxia-inducible factor-1α (HIF-1α) exacerbates tissue damage by driving pro-inflammatory signaling, whereas exosomes derived from BMSCs can restore NP homeostasis by downregulating HIF-1α and inflammatory cytokines (Su et al., 2024).3)During polarization, M1 macrophages exhibit a compensatory increase in OXPHOS activity, generating excessive ATP and ROS to sustain their pro-inflammatory phenotype. This metabolic feature provides a theoretical basis for utilizing nanoparticles to selectively eliminate ROS and block M1 polarization (Li et al., 2022; Wang DK. et al., 2022; Yang W. et al., 2024). Therapeutic strategies targeting these mechanisms have yielded promising results. The limonoid compound Nimbolide has been found to activate the SIRT1 signaling axis, thereby modulating cholesterol metabolism and inflammatory pathways in a dual manner—suppressing M1 polarization while promoting M2 polarization, ultimately fostering a regenerative microenvironment conducive to ECM synthesis (Teng et al., 2023). Additionally, melatonin has been shown to inhibit M1 polarization and alleviate disc degeneration by upregulating SIRT1 expression and modulating the SIRT1/Notch pathway (Dou et al., 2023). These findings suggest that targeting the mitochondrial metabolism-immune regulation network may offer a novel therapeutic approach for IDD.

Given the shared mechanisms underlying OP and IDD, future research in osteomyelitis treatment should focus on the following aspects:1)deciphering the intricate interplay between macrophage polarization and mitochondrial metabolism; 2)developing novel therapies targeting metabolic-immune interaction nodes, such as SIRT agonists combined with ROS scavengers; 3)optimizing biomimetic delivery systems to enable precise interventions at the lesion site. These strategies hold promise for innovative approaches to counteract the progression of bone metabolic disorders.

## 6 Discussion and perspectives

### 6.1 Mechanistic insights and technological integration: from static descriptions to dynamic modulation

The hypoxic characteristics of the osteomyelitis pathological microenvironment, along with biofilm formation, serve as key drivers of metabolic imbalance, profoundly impacting mitochondrial metabolic homeostasis and exacerbating macrophage polarization imbalance. Specifically, local hypoxia stabilizes hypoxia-inducible factor-1α (HIF-1α), shifting macrophage metabolism toward glycolysis while simultaneously impairing OXPHOS through mitochondrial protein lactylation, leading to the accumulation of pro-inflammatory lactate (Chen W. et al., 2022). Moreover, *S.aureus* lipoproteins induce a glycolytic shift and mtROS burst in human monocyte-derived macrophages, characterized by reduced oxygen consumption, lactate accumulation, and a decrease in pH. This metabolic reprogramming disrupts cellular homeostasis in bone, cartilage, and other infected tissues, and mediates lipoprotein‐driven bone erosion (Nguyen et al., 2023).In infectious or inflammatory microenvironments, macrophages can adopt a “metabolic hybrid” phenotype characterized by concurrent glycolysis and OXPHOS. This hybrid state may be driven by local metabolite gradients—such as the succinate/itaconate ratio—suggesting that these metabolites dynamically regulate macrophage polarization. Recent advances have transformed our “static” view of macrophage mitochondrial fission–fusion into a dynamic, high-resolution understanding. First, in humanized bone samples, hypoxia-induced lactate accumulation in osteomyelitis lesions was shown to regulate inflammatory gene expression via histone H3K18 lactylation (Zhang et al., 2019). Second, spatial metabolomics now enables high-resolution mapping of metabolite distributions in tumors and bone marrow, linking succinate/itaconate gradients to macrophage polarization zones (Kriegsmann et al., 2015). Third, metabolic imaging techniques—such as NADH/FAD fluorescence lifetime imaging (FLIM)—can monitor the ratio of reduced to oxidized cofactors *in vivo* or in tissue sections, allowing real-time discrimination of M1 versus M2 macrophage phenotypes (Blacker et al., 2014).Integrating these technologies not only reveals the spatiotemporal dynamics of mitochondrial metabolic reprogramming but also offers new avenues for precisely targeting macrophage function and developing tailored therapies. Future research should focus on metabolic-microenvironment interventions that dynamically reprogram macrophage polarization, disrupt the interplay between pathological features and metabolic dysregulation, and identify novel immunometabolic targets for osteomyelitis treatment.

### 6.2 Targeted therapeutic strategies: from single-pathway interventions to metabolic-immune synergistic modulation

Although therapeutic strategies targeting mitochondrial metabolism have shown promise in preclinical models, their clinical translation remains challenging due to insufficient specificity and pathogen immune evasion. For instance, while metformin promotes M2 polarization via the AMPK/mTOR pathway, systemic administration may disrupt normal immune surveillance, further complicating pathogen clearance in chronic osteomyelitis. In contrast, nanocarrier-based local drug delivery offers a novel direction for osteomyelitis treatment by achieving a “metabolic regulation–antimicrobial–osteogenic” synergistic effect through sustained release and immune microenvironment remodeling. Recent studies have introduced the concept of “metabolic immune checkpoints,” highlighting how pathogens hijack the host succinate-HIF-1α axis to suppress mitophagy and maintain a pro-inflammatory milieu. During persistent infections, pathogens may also evade immune attack by inducing PD-L1 expression. This raises intriguing questions about whether the combined use of SDH inhibitors and PD-1 blockers could enhance antibiotic efficacy in osteomyelitis treatment—an avenue that warrants further exploration. Additionally, spatiotemporally controlled drug delivery systems, such as ROS/pH dual-responsive hydrogels, may offer precise targeting while overcoming the immune tolerance barriers associated with chronic infections. In the osteomyelitis microenvironment, macrophage mitochondrial dynamics are regulated not only by intrinsic metabolic cues but also by signals from other cell types. During early infection, neutrophil‐derived extracellular traps (NETs) and elastase trigger mtROS bursts that activate the NLRP3 inflammasome in macrophages, reinforcing M1 polarization (Singh et al., 2023). Simultaneously, IFN-γ secreted by Th1 cells promotes macrophage OXPHOS and mtROS production to sustain bactericidal M1 functions, whereas IL-4 and IL-10 from Th2 and regulatory T cells activate the STAT6/SIRT1 pathway to enhance mitochondrial biogenesis and drive an M2 reparative phenotype (Viola et al., 2019; Tall and Westerterp, 2019). Additionally, RANKL released by osteoblasts and IL-6 and TGF-β from bone marrow mesenchymal stem cells modulate macrophage mitochondrial metabolism and polarization, thereby influencing the balance between inflammation and repair. The integration of metabolic interventions with cell-based therapies also presents a promising strategy to reprogram the osteomyelitis microenvironment and enhance therapeutic outcomes. Further investigations into these metabolic-immune co-regulation strategies may pave the way for innovative and more effective osteomyelitis treatments.

### 6.3 Clinical translation and biomarkers: from basic research to precision medicine

The successful clinical translation of metabolic regulation strategies for osteomyelitis hinges on addressing two key challenges: 1)Patient Stratification Systems: Mitochondrial metabolic biomarkers—such as plasma succinate levels and mitochondrial DNA copy number—hold potential as indicators for osteomyelitis classification and prognosis. For instance, chronic inflammation patients often exhibit significantly elevated serum succinate levels compared to acute-phase patients, with a positive correlation to the M1/M2 macrophage ratio. New evidence indicates that mitochondrial dysfunction leaves measurable signatures in biological fluids, which may aid in the diagnosis and prognosis of osteomyelitis. Circulating cell-free mitochondrial DNA (ccf-mtDNA)—a damage-associated molecular pattern released from injured cells—is elevated in inflammatory bone diseases; although not yet reported in osteomyelitis patients, plasma ccf-mtDNA is similarly increased in osteoarthritis and correlates with disease severity (Wu YL. et al., 2024). In osteomyelitis patient samples, oxidative stress markers such as malondialdehyde (MDA) and 8-hydroxy-2′-deoxyguanosine (8-OHdG) are significantly elevated, reflecting excessive mtROS production and DNA damage (Özkan et al., 2021). Tricarboxylic acid (TCA) cycle metabolites—particularly succinate and two-oxoglutarate—show altered serum levels in mouse models of osteomyelitis and associate with bacterial burden (Isogai et al., 2020). Together, these biomarkers—ccf-mtDNA, ROS adducts, and mitochondrial metabolites—offer a non-invasive window into mitochondrial health and hold promise as diagnostic or prognostic indicators in osteomyelitis. Notably, acute and chronic osteomyelitis patients display distinct macrophage polarization states and metabolic profiles, posing substantial challenges for both fundamental research and clinical treatment. Therefore, it is crucial to establish individualized research strategies tailored to different osteomyelitis stages. 2)Model Development: Given the complexity of bone metabolism, long study durations, and technical difficulties, modeling osteomyelitis remains a significant challenge. While existing animal models—such as the murine tibial drill-hole infection model—effectively simulate acute infections, they fail to fully recapitulate the metabolic remodeling characteristics of chronic osteomyelitis. 3D Emerging technologies like 3D bioprinting offer promising applications in preclinical research by enabling functional tissue models for drug screening and disease modeling (Ma et al., 2018). Although several studies have utilized 3D-printed models to construct bone infection environments, they often lack in-depth representation of the immune microenvironment. Organoids, engineered using tissue engineering principles to mimic complex biological functions *in vitro*, could serve as an advanced platform for studying osteomyelitis pathophysiology (Chen et al., 2022d). Combining bone organoid technology with 3D-printed infected bone models may allow for a more precise simulation of the intricate pathological microenvironment of osteomyelitis. Moreover, next-generation multi-omics and imaging technologies offer unprecedented spatial and cellular resolution for dissecting macrophage function and mitochondrial dynamics in the osteomyelitis microenvironment. Single-cell multi-omics can simultaneously profile the transcriptome, epigenome, and mitochondrial genotype at single-cell resolution, revealing how metabolic gene expression and mtDNA variations in distinct macrophage subsets influence their polarization (Lareau et al., 2023). Spatial transcriptomics preserves tissue architecture, enabling the localization of macrophage clusters expressing mitochondrial biogenesis and OXPHOS genes within bone lesions and elucidating their spatial interactions with osteoblasts and stromal cells (Feng Y. et al., 2023). High-resolution metabolic imaging—such as FLIM of lactate/NADH autofluorescence and two-photon MitoSOX microscopy—can monitor mitochondrial fusion/fission events and ROS dynamics in real time *in vivo* or in tissue sections, providing direct visual evidence of energy metabolism shifts during macrophage polarization (Hu et al., 2025). The integrated application of these technologies will deepen our understanding of the immunometabolic networks governing macrophage behavior in osteomyelitis and inform the development of precision interventions. Notably, several metabolism‐modulating agents that are already clinically approved or in trials directly target mitochondrial pathways and hold promise for osteomyelitis therapy. Metformin, an AMPK activator and mitochondrial complex I inhibitor widely used in type 2 diabetes, also improves arthritis by activating AMPK and suppressing inflammatory cytokines (Kim et al., 2022; Chen et al., 2020). In sepsis models and clinical observations, metformin reduces macrophage TNF-α and IL-6 secretion and alleviates immunosuppression‐associated dysfunction, highlighting its potential in acute inflammation regulation (Wang et al., 2024c; Ismail Hassan et al., 2020). Dimethyl fumarate (Tecfidera) activates Nrf2-dependent antioxidant programs, promoting mitochondrial biogenesis and inhibiting pro‐inflammatory cytokine release, and is under investigation for inflammatory bone diseases (Gao et al., 2022; Hayashi et al., 2017). The mitochondria‐targeted antioxidant MitoQ was well tolerated in a Phase II trial in chronic hepatitis C patients, significantly lowering ALT levels and reducing necroinflammatory liver damage, likely via mtROS scavenging (Murray et al., 2022; Gane et al., 2010). Additionally, the peptide elamipretide (SS-31), in a randomized crossover Phase II trial for primary mitochondrial myopathy, improved 6-min walk distance after 5 days of dosing without serious adverse events, suggesting enhanced mitochondrial function and cellular bioenergetics (Karaa et al., 2023; Karaa et al., 2018). Integrating these mitochondrial modulators into osteomyelitis regimens could recalibrate macrophage energy metabolism, optimize the immune–repair balance, and ultimately improve clinical outcomes. Moving forward, fostering interdisciplinary collaborations among metabolic biology, bioengineering, and clinical medicine will be essential for establishing a comprehensive research paradigm—spanning mechanistic investigation, target identification, and clinical validation—to ultimately achieve personalized metabolic-immune therapy for osteomyelitis.

## 7 Conclusion

In this systematic review, we have comprehensively explored the molecular mechanisms by which mitochondrial metabolism regulates macrophage polarization and, for the first time, systematically elucidated its critical role in the pathogenesis and progression of osteomyelitis. Additionally, we have examined the broader implications of mitochondrial metabolic regulation in other orthopedic diseases and their underlying mechanisms.

Our findings highlight the significant potential of mitochondrial metabolism in modulating inflammation and promoting bone healing in osteomyelitis, providing a strong theoretical foundation for the development of novel therapeutic strategies targeting mitochondrial metabolism. Notably, targeting mitochondrial metabolism to modulate macrophage polarization may represent a promising avenue for osteomyelitis treatment. This discovery not only deepens our understanding of osteomyelitis pathogenesis but also offers valuable insights for the future development of precision and personalized therapies for this challenging condition.

## Glossary

AMP: adenosine monophosphate
AMPK: AMP-activated protein kinase
Arg1: arginase-1
ATP: adenosine triphosphate
BMP-2: bone morphogenetic protein-2
BMSCs: bone marrow mesenchymal stem cells
cAMP: cyclic adenosine monophosphate
cGAS: cyclic GMP-AMP synthase
cGMP: cyclic guanosine monophosphate
CHI3L1: chitinase 3-like protein 1
Cmpk2: cytidine monophosphate kinase 2
CPT1A: carnitine palmitoyltransferase1A
CTSK: cathepsin K
DAMPs: damage-associated molecular patterns
DRP1: dynamin-related protein 1
ECM: extracellular matrix
ERK: extracellular regulated protein kinases
FAO: fatty acid oxidation
FIS1: fission protein 1
GLUT1: glucose transporter 1
GPX4: glutathione peroxidase 4
GTP: guanosine triphosphate
HK2: hexokinase 2
HIF-1α: hypoxia-inducible factor-1α
HO-1: heme oxygenase 1
IDD: intervertebral disc degeneration
IFN-γ: interferon gamma
IGF-1: insulin-like growth factor-1
IL-1β: interleukin-1beta
IL-4: interleukin-4
IL-6: interleukin-6
IL-10: interleukin-10
iNOS: inducible nitric oxide synthase
JAK: Janus kinase
LDHA: lactate dehydrogenas
LMWF: low-molecular-weight fucoidan
LPS: lipopolysaccharide
MAP3K1: mitogen-activated protein kinase kinase kinase 1
MFN1: mitofusin 1
MFN2: mitofusin 2
MIP-1α: macrophage inflammatory protein-1α
MMP: mitochondrial membrane potential
MRSA: methicillin-resistant *Staphylococcus aureus*
mTOR: mammalian target of rapamycin
mTORC1: mammalian target of rapamycin complex 1
mtROS: mitochondrial reactive oxygen species
NAC: N-acetyl-L-cysteine
NF-κB: nuclear factor kappa-B
NLRP3: NOD-like receptor family pyrin domain containing 3
NOD: Nucleotide-binding oligomerization domain
NP: nucleus pulposus
Nrf2: nuclear factor erythroid 2-related factor 2
NO: nitric oxide
OA: osteoarthritis
OP: osteoporosis
OPG: osteoprotegerin
OXPHOS: oxidative phosphorylation
PDH: pyruvate dehydrogenase
PDK1: pyruvate dehydrogenase kinase 1
PD-1/PD-L1: programmed death 1/programmed cell death ligand 1
PGC-1α: peroxisome proliferator-activated receptor gamma coactivator-1 alpha
PINK1: PTEN induced putative kinase 1
PKG: protein kinase G
PPARγ: peroxisome proliferator-activated receptor gamma
PTEN: phosphatase and tensin homolog deleted on chromosome ten
PTO: post-traumatic osteomyelitis
RA: rheumatoid arthritis
ROS: reactive oxygen species
*S.aureus*: *Staphylococcus aureus*
SCVs: small colony variants
SDH: succinate dehydrogenase
SIRT3: sirtuin-3
SOD2: superoxide dismutase 2
STAT: signal transducer and activator of transcription
STING: stimulator of interferon genes
TCA: tricarboxylic acid
TNF-α: tumour necrosis factor-alpha
TRAP: tartrate-resistant acid phosphatase

## References

[B1] Andrieux P. Chevillard C. Cunha-Neto E. Nunes J. P. S. (2021). Mitochondria as a cellular hub in infection and inflammation. Int. J. Mol. Sci. 22 (21), 11338. 10.3390/ijms222111338 34768767 PMC8583510

[B2] Bai L. Liu Y. Zhang X. Chen P. Hang R. Xiao Y. (2023). Osteoporosis remission via an anti-inflammaging effect by icariin activated autophagy. Biomaterials 297, 122125. 10.1016/j.biomaterials.2023.122125 37058900

[B3] Ballard A. Zeng R. Zarei A. Shao C. Cox L. Yan H. (2020). The tethering function of mitofusin2 controls osteoclast differentiation by modulating the Ca(2+)-NFATc1 axis. J. Biol. Chem. 295 (19), 6629–6640. 10.1074/jbc.RA119.012023 32165499 PMC7212632

[B4] Blacker T. S. Mann Z. F. Gale J. E. Ziegler M. Bain A. J. Szabadkai G. (2014). Separating NADH and NADPH fluorescence in live cells and tissues using FLIM. Nat. Commun. 5, 3936. 10.1038/ncomms4936 24874098 PMC4046109

[B5] Cen M. Ouyang W. Zhang W. Yang L. Lin X. Dai M. (2021). MitoQ protects against hyperpermeability of endothelium barrier in acute lung injury via a Nrf2-dependent mechanism. Redox Biol. 41, 101936. 10.1016/j.redox.2021.101936 33752110 PMC8005834

[B6] Cerutti R. Pirinen E. Lamperti C. Marchet S. Sauve A. A. Li W. (2014). NAD(+)-dependent activation of Sirt1 corrects the phenotype in a mouse model of mitochondrial disease. Cell Metab. 19 (6), 1042–1049. 10.1016/j.cmet.2014.04.001 24814483 PMC4051987

[B7] Chatsirisupachai A. Muanjumpon P. Jeayeng S. Onkoksong T. Pluempreecha M. Soingam T. (2024). Calcitriol/vitamin D receptor system alleviates PM2.5-induced human bronchial epithelial damage through upregulating mitochondrial bioenergetics in association with regulation of HIF-1α/PGC-1α signaling. Environ. Toxicol. Pharmacol. 111, 104568. 10.1016/j.etap.2024.104568 39307374

[B8] Chen D. Shen J. Zhao W. Wang T. Han L. Hamilton J. L. (2017). Osteoarthritis: toward a comprehensive understanding of pathological mechanism. Bone Res. 5, 16044. 10.1038/boneres.2016.44 28149655 PMC5240031

[B9] Chen H. Zhang J. He Y. Lv Z. Liang Z. Chen J. (2022a). Exploring the role of *Staphylococcus aureus* in inflammatory diseases. Toxins (Basel). 14 (7), 464. 10.3390/toxins14070464 35878202 PMC9318596

[B10] Chen M. Wang J. Zhang P. Jiang Z. Chen S. Liang S. (2025a). Low molecular weight fucoidan induces M2 macrophage polarization to attenuate inflammation through activation of the AMPK/mTOR autophagy pathway. Eur. J. Pharmacol. 986, 177134. 10.1016/j.ejphar.2024.177134 39547407

[B11] Chen Q. Qian Q. Xu H. Zhou H. Chen L. Shao N. (2024c). Mitochondrial-targeted metal-phenolic nanoparticles to attenuate intervertebral disc degeneration: alleviating oxidative stress and mitochondrial dysfunction. ACS Nano 18 (12), 8885–8905. 10.1021/acsnano.3c12163 38465890

[B12] Chen S. Chen X. Geng Z. Su J. (2022d). The horizon of bone organoid: a perspective on construction and application. Bioact. Mater 18, 15–25. 10.1016/j.bioactmat.2022.01.048 35387160 PMC8961298

[B13] Chen S. Li Y. Fu S. Li Y. Wang C. Sun P. (2022b). Melatonin alleviates arginine vasopressin-induced cardiomyocyte apoptosis via increasing Mst1-Nrf2 pathway activity to reduce oxidative stress. Biochem. Pharmacol. 206, 115265. 10.1016/j.bcp.2022.115265 36183803

[B14] Chen W. Wu P. Yu F. Luo G. Qing L. Tang J. (2022c). HIF-1α regulates bone homeostasis and angiogenesis, participating in the occurrence of bone metabolic diseases. Cells 11 (22), 3552. 10.3390/cells11223552 36428981 PMC9688488

[B15] Chen Y. Dong J. Li J. Li J. Lu Y. Dong W. (2024a). Engineered macrophage-derived exosomes via click chemistry for the treatment of osteomyelitis. J. Mater Chem. B 12 (41), 10593–10604. 10.1039/d4tb01346h 39315933

[B16] Chen Y. Li C. Jia J. Jiang Y. Zhang P. Cheng C. (2025b). COX-2 inhibition as a therapeutic strategy for bone loss in *Staphylococcus aureus* osteomyelitis. Mol. Med. 31 (1), 177. 10.1186/s10020-025-01202-9 40335904 PMC12057237

[B17] Chen Y. Liu Z. Lin Z. Lu M. Fu Y. Liu G. (2023). The effect of *Staphylococcus aureus* on innate and adaptive immunity and potential immunotherapy for S. aureus-induced osteomyelitis. Front. Immunol. 14, 1219895. 10.3389/fimmu.2023.1219895 37744377 PMC10517662

[B18] Chen Y. Qiu F. Yu B. Chen Y. Zuo F. Zhu X. (2020). Metformin, an AMPK activator, inhibits activation of FLSs but promotes HAPLN1 secretion. Mol. Ther. Methods Clin. Dev. 17, 1202–1214. 10.1016/j.omtm.2020.05.008 32518807 PMC7275116

[B19] Chen Z. Pang Q. Zhan J. Liu J. Zhao W. Dong L. (2024b). MSCs-derived ECM functionalized hydrogel regulates macrophage reprogramming for osteoarthritis treatment by improving mitochondrial function and energy metabolism. Mater Today Bio 29, 101340. 10.1016/j.mtbio.2024.101340 PMC11617891 39640869

[B20] Cheng J. W. Yu Y. Zong S. Y. Cai W. W. Wang Y. Song Y. N. (2023). Berberine ameliorates collagen-induced arthritis in mice by restoring macrophage polarization via AMPK/mTORC1 pathway switching glycolytic reprogramming. Int. Immunopharmacol. 124 (Pt B), 111024. 10.1016/j.intimp.2023.111024 37827054

[B21] Clayton S. A. MacDonald L. Kurowska-Stolarska M. Clark A. R. (2021). Mitochondria as key players in the pathogenesis and treatment of rheumatoid arthritis. Front. Immunol. 12, 673916. 10.3389/fimmu.2021.673916 33995417 PMC8118696

[B22] Cutolo M. Campitiello R. Gotelli E. Soldano S. (2022). The role of M1/M2 macrophage polarization in rheumatoid arthritis synovitis. Front. Immunol. 13, 867260. 10.3389/fimmu.2022.867260 35663975 PMC9161083

[B23] Dai Y. Yi X. Huang Y. Qian K. Huang L. Hu J. (2024). miR-345-3p modulates M1/M2 macrophage polarization to inhibit inflammation in bone infection via targeting MAP3K1 and NF-κB pathway. J. Immunol. 212 (5), 844–854. 10.4049/jimmunol.2300561 38231123

[B24] Dapunt U. Maurer S. Giese T. Gaida M. M. Hänsch G. M. (2014). The macrophage inflammatory proteins MIP1α (CCL3) and MIP2α (CXCL2) in implant-associated osteomyelitis: linking inflammation to bone degradation. Mediat. Inflamm. 2014, 728619. 10.1155/2014/728619 PMC3984830 24795505

[B25] Dong G. Xu N. Wang M. Zhao Y. Jiang F. Bu H. (2021). Anthocyanin extract from purple sweet potato exacerbate mitophagy to ameliorate pyroptosis in *Klebsiella pneumoniae* infection. Int. J. Mol. Sci. 22 (21), 11422. 10.3390/ijms222111422 34768852 PMC8583717

[B26] Dong T. Chen X. Xu H. Song Y. Wang H. Gao Y. (2022). Mitochondrial metabolism mediated macrophage polarization in chronic lung diseases. Pharmacol. Ther. 239, 108208. 10.1016/j.pharmthera.2022.108208 35569553

[B27] Dou X. Luo Q. Xie L. Zhou X. Song C. Liu M. (2023). Medical prospect of melatonin in the intervertebral disc degeneration through inhibiting M1-type macrophage polarization via SIRT1/notch signaling pathway. Biomedicines 11 (6), 1615. 10.3390/biomedicines11061615 37371708 PMC10296002

[B28] Fadok V. A. Bratton D. L. Konowal A. Freed P. W. Westcott J. Y. Henson P. M. (1998). Macrophages that have ingested apoptotic cells *in vitro* inhibit proinflammatory cytokine production through autocrine/paracrine mechanisms involving TGF-beta, PGE2, and PAF. J. Clin. Invest 101 (4), 890–898. 10.1172/JCI1112 9466984 PMC508637

[B29] Fan N. Zhang X. Zhao W. Zhao J. Luo D. Sun Y. (2022). Covalent inhibition of pyruvate kinase M2 reprograms metabolic and inflammatory pathways in hepatic macrophages against non-alcoholic fatty liver disease. Int. J. Biol. Sci. 18 (14), 5260–5275. 10.7150/ijbs.73890 36147457 PMC9461663

[B30] Feng X. Liu Z. Su Y. Lian H. Gao Y. Zhao J. (2023b). Tussilagone inhibits osteoclastogenesis by modulating mitochondrial function and ROS production involved Nrf2 activation. Biochem. Pharmacol. 218, 115895. 10.1016/j.bcp.2023.115895 38084677

[B31] Feng Y. Wang S. Xie J. Ding B. Wang M. Zhang P. (2023c). Spatial transcriptomics reveals heterogeneity of macrophages in the tumor microenvironment of granulomatous slack skin. J. Pathol. 261 (1), 105–119. 10.1002/path.6151 37550813

[B32] Feng Z. Jing Z. Li Q. Chu L. Jiang Y. Zhang X. (2023a). Exosomal STIMATE derived from type II alveolar epithelial cells controls metabolic reprogramming of tissue-resident alveolar macrophages. Theranostics 13 (3), 991–1009. 10.7150/thno.82552 36793853 PMC9925314

[B33] Ferver A. Greene E. Wideman R. Dridi S. (2021). Evidence of mitochondrial dysfunction in bacterial chondronecrosis with osteomyelitis-affected broilers. Front. Vet. Sci. 8, 640901. 10.3389/fvets.2021.640901 33634182 PMC7902039

[B34] Fu J. Li Y. Zhang Y. Liang Y. Zheng Y. Li Z. (2021). An engineered pseudo-macrophage for rapid treatment of bacteria-infected osteomyelitis via microwave-excited anti-infection and immunoregulation. Adv. Mater 33 (41), e2102926. 10.1002/adma.202102926 34396595

[B35] Fu W. Fang Y. Wang T. Lu Q. Wu J. Yang Q. (2024). Low-protein diet inhibits the synovial tissue macrophage pro-inflammatory polarization via NRF2/SIRT3/SOD2/ROS pathway in K/BxN rheumatoid arthritis mice. Inflammation. 10.1007/s10753-024-02145-9 39292325

[B36] Fu Z. J. Wang Z. Y. Xu L. Chen X. H. Li X. X. Liao W. T. (2020). HIF-1α-BNIP3-mediated mitophagy in tubular cells protects against renal ischemia/reperfusion injury. Redox Biol. 36, 101671. 10.1016/j.redox.2020.101671 32829253 PMC7452120

[B37] Fuhrmann D. C. Wittig I. Brüne B. (2019). TMEM126B deficiency reduces mitochondrial SDH oxidation by LPS, attenuating HIF-1α stabilization and IL-1β expression. Redox Biol. 20, 204–216. 10.1016/j.redox.2018.10.007 30368040 PMC6202876

[B38] Gane E. J. Weilert F. Orr D. W. Keogh G. F. Gibson M. Lockhart M. M. (2010). The mitochondria-targeted anti-oxidant mitoquinone decreases liver damage in a phase II study of hepatitis C patients. Liver Int. 30 (7), 1019–1026. 10.1111/j.1478-3231.2010.02250.x 20492507

[B39] Gao S. J. Li D. Y. Liu D. Q. Sun J. Zhang L. Q. Wu J. Y. (2022). Dimethyl fumarate attenuates pain behaviors in osteoarthritis rats via induction of nrf2-mediated mitochondrial biogenesis. Mol. Pain 18, 17448069221124920. 10.1177/17448069221124920 36065971 PMC9478692

[B40] Ge G. Bai J. Wang Q. Liang X. Tao H. Chen H. (2022). Punicalagin ameliorates collagen-induced arthritis by downregulating M1 macrophage and pyroptosis via NF-κB signaling pathway. Sci. China Life Sci. 65 (3), 588–603. 10.1007/s11427-020-1939-1 34125371

[B41] Geng Q. Xu J. Cao X. Wang Z. Jiao Y. Diao W. (2024). PPARG-mediated autophagy activation alleviates inflammation in rheumatoid arthritis. J. Autoimmun. 146, 103214. 10.1016/j.jaut.2024.103214 38648706

[B42] Gobelli D. Serrano-Lorenzo P. Esteban-Amo M. J. Serna J. Pérez-García M. T. Orduña A. (2023). The mitochondrial succinate dehydrogenase complex controls the STAT3-IL-10 pathway in inflammatory macrophages. iScience 26 (8), 107473. 10.1016/j.isci.2023.107473 37575201 PMC10416071

[B43] Gobert A. P. Wilson K. T. (2012). Editorial: orchestration of macrophage polarization by polyamines. J. Leukoc. Biol. 91 (5), 677–679. 10.1189/jlb.0112047 22547131 PMC3336771

[B44] Granata V. Possetti V. Parente R. Bottazzi B. Inforzato A. Sobacchi C. (2022). The osteoblast secretome in *Staphylococcus aureus* osteomyelitis. Front. Immunol. 13, 1048505. 10.3389/fimmu.2022.1048505 36483565 PMC9723341

[B45] Gu C. Chen M. Li X. Geng D. Wang C. (2023). MAGL regulates synovial macrophage polarization vis inhibition of mitophagy in osteoarthritic pain. Mol. Med. Rep. 27 (6), 117. 10.3892/mmr.2023.13004 37144506 PMC10172810

[B46] Gu L. Larson-Casey J. L. Carter A. B. (2017). Macrophages utilize the mitochondrial calcium uniporter for profibrotic polarization. Faseb J. 31 (7), 3072–3083. 10.1096/fj.201601371R 28351840 PMC5471522

[B47] Guo J. Tang X. Deng P. Hui H. Chen B. An J. (2024). Interleukin-4 from curcumin-activated OECs emerges as a central modulator for increasing M2 polarization of microglia/macrophage in OEC anti-inflammatory activity for functional repair of spinal cord injury. Cell Commun. Signal 22 (1), 162. 10.1186/s12964-024-01539-4 38448976 PMC10916222

[B48] Guo Y. Xie C. Li X. Yang J. Yu T. Zhang R. (2017). Succinate and its G-protein-coupled receptor stimulates osteoclastogenesis. Nat. Commun. 8, 15621. 10.1038/ncomms15621 28561074 PMC5460032

[B49] Hayashi G. Jasoliya M. Sahdeo S. Saccà F. Pane C. Filla A. (2017). Dimethyl fumarate mediates Nrf2-dependent mitochondrial biogenesis in mice and humans. Hum. Mol. Genet. 26 (15), 2864–2873. 10.1093/hmg/ddx167 28460056 PMC6251607

[B50] He B. Wang X. Zhu J. Kong B. Wei L. Jin Y. (2019). Autophagy protects murine macrophages from β-cypermethrin-induced mitochondrial dysfunction and cytotoxicity via the reduction of oxidation stress. Environ. Pollut. 250, 416–425. 10.1016/j.envpol.2019.04.044 31026688

[B51] He C. Chen P. Ning L. Huang X. Sun H. Wang Y. (2025). Inhibition of mitochondrial succinate dehydrogenase with dimethyl malonate promotes M2 macrophage polarization by enhancing STAT6 activation. Inflammation. 10.1007/s10753-024-02207-y 39806091

[B52] He X. X. Huang Y. J. Hu C. L. Xu Q. Q. Wei Q. J. (2024). Songorine modulates macrophage polarization and metabolic reprogramming to alleviate inflammation in osteoarthritis. Front. Immunol. 15, 1344949. 10.3389/fimmu.2024.1344949 38415250 PMC10896988

[B53] Hu K. Shang Z. Yang X. Zhang Y. Cao L. (2023). Macrophage polarization and the regulation of bone immunity in bone homeostasis. J. Inflamm. Res. 16, 3563–3580. 10.2147/JIR.S423819 37636272 PMC10460180

[B54] Hu L. West A. P. Walsh A. J. (2025). Optical metabolic imaging identifies metabolic shifts and mitochondria heterogeneity in POLG mutator macrophages. bioRxiv, 2025.01.13.632822. 10.1101/2025.01.13.632822

[B55] Huang L. Wang J. Yu J. Bian M. Xiang X. Han G. (2024a). Picein alleviates oxidative stress and promotes bone regeneration in osteoporotic bone defect by inhibiting ferroptosis via Nrf2/HO-1/GPX4 pathway. Environ. Toxicol. 39 (7), 4066–4085. 10.1002/tox.24239 38727095

[B56] Huang X. Deng Y. Xiao J. Wang H. Yang Q. Cao Z. (2024b). Genetically engineered M2-like macrophage-derived exosomes for P. gingivalis-suppressed cementum regeneration: from mechanism to therapy. Bioact. Mater 32, 473–487. 10.1016/j.bioactmat.2023.10.009 37965240 PMC10640966

[B57] Ismail Hassan F. Didari T. Khan F. Niaz K. Mojtahedzadeh M. Abdollahi M. (2020). A review on the protective effects of metformin in sepsis-induced organ failure. Cell J. 21 (4), 363–370. 10.22074/cellj.2020.6286 31376317 PMC6722446

[B58] Isogai N. Shiono Y. Kuramoto T. Yoshioka K. Ishihama H. Funao H. (2020). Potential osteomyelitis biomarkers identified by plasma metabolome analysis in mice. Sci. Rep. 10 (1), 839. 10.1038/s41598-020-57619-1 31964942 PMC6972943

[B59] Jeong S. Seong J. H. Kang J. H. Lee D. S. Yim M. (2021). Dynamin-related protein 1 positively regulates osteoclast differentiation and bone loss. FEBS Lett. 595 (1), 58–67. 10.1002/1873-3468.13963 33084048

[B60] Jia N. Gao Y. Li M. Liang Y. Li Y. Lin Y. (2023). Metabolic reprogramming of proinflammatory macrophages by target delivered roburic acid effectively ameliorates rheumatoid arthritis symptoms. Signal Transduct. Target Ther. 8 (1), 280. 10.1038/s41392-023-01499-0 37500654 PMC10374631

[B61] Jin K. Ma Y. Manrique-Caballero C. L. Li H. Emlet D. R. Li S. (2020). Activation of AMP-activated protein kinase during sepsis/inflammation improves survival by preserving cellular metabolic fitness. Faseb J. 34 (5), 7036–7057. 10.1096/fj.201901900R 32246808 PMC11956121

[B62] Jin M. Wu X. Hu J. Chen Y. Yang B. Cheng C. (2024). EGFR-MEK1/2 cascade negatively regulates bactericidal function of bone marrow macrophages in mice with *Staphylococcus aureus* osteomyelitis. PLoS Pathog. 20 (8), e1012437. 10.1371/journal.ppat.1012437 39102432 PMC11326603

[B63] Jin Z. Wei W. Yang M. Du Y. Wan Y. (2014). Mitochondrial complex I activity suppresses inflammation and enhances bone resorption by shifting macrophage-osteoclast polarization. Cell Metab. 20 (3), 483–498. 10.1016/j.cmet.2014.07.011 25130399 PMC4156549

[B64] Kang M. Huang C. C. Lu Y. Shirazi S. Gajendrareddy P. Ravindran S. (2020). Bone regeneration is mediated by macrophage extracellular vesicles. Bone 141, 115627. 10.1016/j.bone.2020.115627 32891867 PMC8107826

[B65] Karaa A. Bertini E. Carelli V. Cohen B. H. Enns G. M. Falk M. J. (2023). Efficacy and safety of elamipretide in individuals with primary mitochondrial myopathy: the MMPOWER-3 randomized clinical trial. Neurology 101 (3), e238–e252. 10.1212/WNL.0000000000207402 37268435 PMC10382259

[B66] Karaa A. Haas R. Goldstein A. Vockley J. Weaver W. D. Cohen B. H. (2018). Randomized dose-escalation trial of elamipretide in adults with primary mitochondrial myopathy. Neurology 90 (14), e1212–e1221. 10.1212/WNL.0000000000005255 29500292 PMC5890606

[B67] Kavanagh N. Ryan E. J. Widaa A. Sexton G. Fennell J. O'Rourke S. (2018). Staphylococcal osteomyelitis: disease progression, treatment challenges, and future directions. Clin. Microbiol. Rev. 31 (2), e00084-17. 10.1128/CMR.00084-17 PMC5967688 29444953

[B68] Kawano I. Bazila B. Ježek P. Dlasková A. (2023). Mitochondrial dynamics and cristae shape changes during metabolic reprogramming. Antioxid. Redox Signal 39 (10-12), 684–707. 10.1089/ars.2023.0268 37212238

[B69] Kim J. W. Choe J. Y. Park S. H. (2022). Metformin and its therapeutic applications in autoimmune inflammatory rheumatic disease. Korean J. Intern Med. 37 (1), 13–26. 10.3904/kjim.2021.363 34879473 PMC8747910

[B70] Kontogianni G. I. Loukelis K. Bonatti A. F. Batoni E. De Maria C. Vozzi G. (2025). A mechanically stimulated Co-culture in 3-dimensional composite scaffolds promotes osteogenic and anti-osteoclastogenic activity and M2 macrophage polarization. Biomater. Res. 29, 0135. 10.34133/bmr.0135 39911306 PMC11794764

[B71] Koroth J. Buko E. O. Abbott R. Johnson C. P. Ogle B. M. Stone L. S. (2023). Macrophages and intervertebral disc degeneration. Int. J. Mol. Sci. 24 (2), 1367. 10.3390/ijms24021367 36674887 PMC9863885

[B72] Kremers H. M. Nwojo M. E. Ransom J. E. Wood-Wentz C. M. Melton L. J. 3rd Huddleston P. M. 3rd (2015). Trends in the epidemiology of osteomyelitis: a population-based study, 1969 to 2009. J. Bone Jt. Surg. Am. 97 (10), 837–845. 10.2106/JBJS.N.01350 PMC4642868 25995495

[B73] Kriegsmann J. Kriegsmann M. Casadonte R. (2015). MALDI TOF imaging mass spectrometry in clinical pathology: a valuable tool for cancer diagnostics (review). Int. J. Oncol. 46 (3), 893–906. 10.3892/ijo.2014.2788 25482502

[B74] Lareau C. A. Dubois S. M. Buquicchio F. A. Hsieh Y. H. Garg K. Kautz P. (2023). Single-cell multi-omics of mitochondrial DNA disorders reveals dynamics of purifying selection across human immune cells. Nat. Genet. 55 (7), 1198–1209. 10.1038/s41588-023-01433-8 37386249 PMC10548551

[B75] Lew D. P. Waldvogel F. A. (2004). Osteomyelitis. Lancet 364 (9431), 369–379. 10.1016/S0140-6736(04)16727-5 15276398

[B76] Ley K. Laudanna C. Cybulsky M. I. Nourshargh S. (2007). Getting to the site of inflammation: the leukocyte adhesion cascade updated. Nat. Rev. Immunol. 7 (9), 678–689. 10.1038/nri2156 17717539

[B77] Li A. Liu Y. Yu P. Zhang Z. Huang T. Li H. (2025a). Ergothioneine attenuates psoriasis symptoms through modulation of M1/M2 macrophage polarisation via the NF-κB/JAK-STAT3 pathway. Front. Pharmacol. 16, 1521743. 10.3389/fphar.2025.1521743 40046751 PMC11880282

[B78] Li J. Guo T. Li Y. Wang Q. Du Y. Li R. (2024a). Adipose stem cells regulate lipid metabolism by upregulating mitochondrial fatty acid β-oxidation in macrophages to improve the retention rate of transplanted fat. Stem Cell Res. Ther. 15 (1), 328. 10.1186/s13287-024-03953-4 39334483 PMC11438425

[B79] Li K. Chen Y. Lin Y. Zhang G. Su J. Wu X. (2023a). PD-1/PD-L1 blockade is a potent adjuvant in treatment of *Staphylococcus aureus* osteomyelitis in mice. Mol. Ther. 31 (1), 174–192. 10.1016/j.ymthe.2022.09.006 36104974 PMC9840119

[B80] Li M. Niu Y. Tian L. Zhang T. Zhou S. Wang L. (2024b). Astragaloside IV alleviates macrophage senescence and d-galactose-induced bone loss in mice through STING/NF-κB pathway. Int. Immunopharmacol. 129, 111588. 10.1016/j.intimp.2024.111588 38290207

[B81] Li X. Shen H. Zhang M. Teissier V. Huang E. E. Gao Q. (2023b). Glycolytic reprogramming in macrophages and MSCs during inflammation. Front. Immunol. 14, 1199751. 10.3389/fimmu.2023.1199751 37675119 PMC10477714

[B82] Li X. C. Luo S. J. Fan W. Zhou T. L. Tan D. Q. Tan R. X. (2022). Macrophage polarization regulates intervertebral disc degeneration by modulating cell proliferation, inflammation mediator secretion, and extracellular matrix metabolism. Front. Immunol. 13, 922173. 10.3389/fimmu.2022.922173 36059551 PMC9433570

[B83] Li Y. Ai S. Li Y. Ye W. Li R. Xu X. (2025b). The role of natural products targeting macrophage polarization in sepsis-induced lung injury. Chin. Med. 20 (1), 19. 10.1186/s13020-025-01067-4 39910395 PMC11800549

[B84] Li Z. Lu Y. Song J. Han P. Shi H. Yao X. (2025c). An emodin-mediated multifunctional nanoplatform with augmented sonodynamic and immunoregulation for osteomyelitis therapy. J. Colloid Interface Sci. 684 (Pt 2), 122–137. 10.1016/j.jcis.2025.01.094 39823728

[B85] Liu H. Song Y. Wang H. Zhou Y. Xu M. Xian J. (2025). Deciphering the power of resveratrol in mitophagy: from molecular mechanisms to therapeutic applications. Phytother. Res. 39 (3), 1319–1343. 10.1002/ptr.8433 39754508

[B86] Liu J. Zhang X. Hou H. Ouyang J. Dai J. (2024b). Advances in osteoblast and mitochondrial dynamics and their transfer in osteoporosis. J. Cell Mol. Med. 28 (24), e70299. 10.1111/jcmm.70299 39700051 PMC11657594

[B87] Liu K. Zhou X. Fang L. Dong J. Cui L. Li J. (2022). PINK1/parkin-mediated mitophagy alleviates Staphylococcus aureus-induced NLRP3 inflammasome and NF-κB pathway activation in bovine mammary epithelial cells. Int. Immunopharmacol. 112, 109200. 10.1016/j.intimp.2022.109200 36063687

[B88] Liu L. Xiang C. Li T. Zhao Z. Xiao T. Ouyang Z. (2024a). Inhibition of NF-κB and ERK signaling pathways in osteoclasts and M1 macrophage polarization: mechanistic insights into the anti-osteoporotic effects of Pseudolaric acid B. Life Sci. 345, 122592. 10.1016/j.lfs.2024.122592 38554947

[B89] Liu M. Wang J. Chen S. Meng X. Cheng Z. Wang J. (2023). Exploring the effect of Er miao San-containing serum on macrophage polarization through miR-33/NLRP3 pathway. J. Ethnopharmacol. 307, 116178. 10.1016/j.jep.2023.116178 36708884

[B90] Liu P. S. Ho P. C. (2018). Mitochondria: a master regulator in macrophage and T cell immunity. Mitochondrion 41, 45–50. 10.1016/j.mito.2017.11.002 29146487

[B91] Liu Y. Hao R. Lv J. Yuan J. Wang X. Xu C. (2024c). Targeted knockdown of PGAM5 in synovial macrophages efficiently alleviates osteoarthritis. Bone Res. 12 (1), 15. 10.1038/s41413-024-00318-8 38433252 PMC10909856

[B92] Luchkova A. Mata A. Cadenas S. (2024). Nrf2 as a regulator of energy metabolism and mitochondrial function. FEBS Lett. 598 (17), 2092–2105. 10.1002/1873-3468.14993 39118293

[B93] Luo J. Wang J. Zhang J. Sang A. Ye X. Cheng Z. (2022). Nrf2 deficiency exacerbated CLP-induced pulmonary injury and inflammation through autophagy- and NF-κB/PPARγ-Mediated macrophage polarization. Cells 11 (23), 3927. 10.3390/cells11233927 36497185 PMC9735993

[B94] Lv H. Yang M. Yang Y. Tang Z. Guo Y. Zhou J. (2025). Metal ion and antibiotic Co-loaded nanoparticles for combating methicillin-rresistant Staphylococcus aureus-induced osteomyelitis. ACS Nano 19 (5), 5253–5268. 10.1021/acsnano.4c11956 39886847

[B95] Ma X. Liu J. Zhu W. Tang M. Lawrence N. Yu C. (2018). 3D bioprinting of functional tissue models for personalized drug screening and *in vitro* disease modeling. Adv. Drug Deliv. Rev. 132, 235–251. 10.1016/j.addr.2018.06.011 29935988 PMC6226327

[B96] Mao Y. Zhang J. Zhou Q. He X. Zheng Z. Wei Y. (2024). Hypoxia induces mitochondrial protein lactylation to limit oxidative phosphorylation. Cell Res. 34 (1), 13–30. 10.1038/s41422-023-00864-6 38163844 PMC10770133

[B97] Masters E. A. Ricciardi B. F. Bentley K. L. M. Moriarty T. F. Schwarz E. M. Muthukrishnan G. (2022). Skeletal infections: microbial pathogenesis, immunity and clinical management. Nat. Rev. Microbiol. 20 (7), 385–400. 10.1038/s41579-022-00686-0 35169289 PMC8852989

[B98] Mendelsohn D. H. Niedermair T. Walter N. Alt V. Rupp M. Brochhausen C. (2023). Ultrastructural evidence of mitochondrial dysfunction in osteomyelitis patients. Int. J. Mol. Sci. 24 (6), 5709. 10.3390/ijms24065709 36982790 PMC10053973

[B99] Mi B. Chen L. Xiong Y. Yang Y. Panayi A. C. Xue H. (2022). Osteoblast/osteoclast and immune cocktail therapy of an exosome/drug delivery multifunctional hydrogel accelerates fracture repair. ACS Nano 16 (1), 771–782. 10.1021/acsnano.1c08284 34979087

[B100] Mills C. D. (2015). Anatomy of a discovery: m1 and m2 macrophages. Front. Immunol. 6, 212. 10.3389/fimmu.2015.00212 25999950 PMC4419847

[B101] Mouton A. J. Li X. Hall M. E. Hall J. E. (2020). Obesity, hypertension, and cardiac dysfunction: novel roles of immunometabolism in macrophage activation and inflammation. Circ. Res. 126 (6), 789–806. 10.1161/CIRCRESAHA.119.312321 32163341 PMC7255054

[B102] Muñoz J. Akhavan N. S. Mullins A. P. Arjmandi B. H. (2020). Macrophage polarization and osteoporosis: a review. Nutrients 12 (10), 2999. 10.3390/nu12102999 33007863 PMC7601854

[B103] Murray K. O. Berryman-Maciel M. Darvish S. Coppock M. E. You Z. Chonchol M. (2022). Mitochondrial-targeted antioxidant supplementation for improving age-related vascular dysfunction in humans: a study protocol. Front. Physiol. 13, 980783. 10.3389/fphys.2022.980783 36187760 PMC9520456

[B104] Muthukrishnan G. Masters E. A. Daiss J. L. Schwarz E. M. (2019). Mechanisms of immune evasion and bone tissue colonization that make *Staphylococcus aureus* the primary pathogen in osteomyelitis. Curr. Osteoporos. Rep. 17 (6), 395–404. 10.1007/s11914-019-00548-4 31721069 PMC7344867

[B105] Nan D. Rao C. Tang Z. Yang W. Wu P. Chen J. (2024). Burkholderia pseudomallei BipD modulates host mitophagy to evade killing. Nat. Commun. 15 (1), 4740. 10.1038/s41467-024-48824-x 38834545 PMC11150414

[B106] Nguyen M. T. Hu Z. Mohammad M. Schöttler H. Niemann S. Schultz M. (2023). Bacterial lipoproteins shift cellular metabolism to glycolysis in macrophages causing bone erosion. Microbiol. Spectr. 11 (3), e0429322. 10.1128/spectrum.04293-22 37191536 PMC10269925

[B107] Nishioku T. Anzai R. Hiramatsu S. Terazono A. Nakao M. Moriyama M. (2023). Lactate dehydrogenase A inhibition prevents RANKL-induced osteoclastogenesis by reducing enhanced glycolysis. J. Pharmacol. Sci. 153 (4), 197–207. 10.1016/j.jphs.2023.09.005 37973217

[B108] Özkan S. Adanaş C. Demir C. Hakan H. (2021). The levels of oxidative DNA damage and some antioxidants in chronic osteomyelitis patients: a cross-sectional study. Int. J. Clin. Pract. 75 (11), e14704. 10.1111/ijcp.14704 34363724

[B109] Peilin W. Ying P. Renyuan W. Zhuoxuan L. Zhenwu Y. Mai Z. (2023). Size-dependent gold nanoparticles induce macrophage M2 polarization and promote intracellular clearance of *Staphylococcus aureus* to alleviate tissue infection. Mater Today Bio 21, 100700. 10.1016/j.mtbio.2023.100700 PMC10338365 37455821

[B110] Pidwill G. R. Gibson J. F. Cole J. Renshaw S. A. Foster S. J. (2020). The role of macrophages in *Staphylococcus aureus* infection. Front. Immunol. 11, 620339. 10.3389/fimmu.2020.620339 33542723 PMC7850989

[B111] Qian L. Zhu Y. Deng C. Liang Z. Chen J. Chen Y. (2024). Peroxisome proliferator-activated receptor gamma coactivator-1 (PGC-1) family in physiological and pathophysiological process and diseases. Signal Transduct. Target Ther. 9 (1), 50. 10.1038/s41392-024-01756-w 38424050 PMC10904817

[B112] Qin Y. Hu C. Jin J. Chao Y. Wang D. Xia F. (2024). Bilobalide ameliorates osteoporosis by influencing the SIRT3/NF-κB axis in osteoclasts and promoting M2 polarization in macrophages. Int. J. Biol. Macromol. 281 (Pt 4), 136504. 10.1016/j.ijbiomac.2024.136504 39395513

[B113] Qing J. Zhang Z. Novák P. Zhao G. Yin K. (2020). Mitochondrial metabolism in regulating macrophage polarization: an emerging regulator of metabolic inflammatory diseases. Acta Biochim. Biophys. Sin. (Shanghai) 52 (9), 917–926. 10.1093/abbs/gmaa081 32785581

[B114] Qiu H. Xiong H. Zheng J. Peng Y. Wang C. Hu Q. (2024). Sr-incorporated bioactive glass remodels the immunological microenvironment by enhancing the mitochondrial function of macrophage via the PI3K/AKT/mTOR signaling pathway. ACS Biomater. Sci. Eng. 10 (6), 3923–3934. 10.1021/acsbiomaterials.4c00228 38766805

[B115] Ren Y. Khan F. A. Pandupuspitasari N. S. Zhang S. (2017). Immune evasion strategies of pathogens in macrophages: the potential for limiting pathogen transmission. Curr. Issues Mol. Biol. 21, 21–40. 10.21775/cimb.021.021 27033743

[B116] Roy S. Wang S. Ullah Z. Hao H. Xu R. Roy J. (2025). Defect-engineered biomimetic piezoelectric nanocomposites with enhanced ROS production, macrophage Re-polarization, and Ca(2+) channel activation for therapy of MRSA-infected wounds and osteomyelitis. Small 21 (10), e2411906. 10.1002/smll.202411906 39887853

[B117] Saxena A. Lopes F. McKay D. M. (2018). Reduced intestinal epithelial mitochondrial function enhances *in vitro* interleukin-8 production in response to commensal *Escherichia coli* . Inflamm. Res. 67 (10), 829–837. 10.1007/s00011-018-1172-5 30030553

[B118] Scozzi D. Cano M. Ma L. Zhou D. Zhu J. H. O'Halloran J. A. (2021). Circulating mitochondrial DNA is an early indicator of severe illness and mortality from COVID-19. JCI Insight 6 (4), e143299. 10.1172/jci.insight.143299 33444289 PMC7934921

[B119] Sha W. Zhao B. Wei H. Yang Y. Yin H. Gao J. (2023). Astragalus polysaccharide ameliorates vascular endothelial dysfunction by stimulating macrophage M2 polarization via potentiating Nrf2/HO-1 signaling pathway. Phytomedicine 112, 154667. 10.1016/j.phymed.2023.154667 36842218

[B120] Singh P. Kumar N. Singh M. Kaur M. Singh G. Narang A. (2023). Neutrophil extracellular traps and NLRP3 inflammasome: a disturbing duo in atherosclerosis, inflammation and atherothrombosis. Vaccines (Basel). 11 (2), 261. 10.3390/vaccines11020261 36851139 PMC9966193

[B121] Soliman E. Elhassanny A. E. M. Malur A. McPeek M. Bell A. Leffler N. (2020). Impaired mitochondrial function of alveolar macrophages in carbon nanotube-induced chronic pulmonary granulomatous disease. Toxicology 445, 152598. 10.1016/j.tox.2020.152598 32976959 PMC7606835

[B122] Soliman W. A. H. Abdelsattar A. M. Hussein A. A. El-Sayed Ellakwa D. Hamed Elmoghazy N. Gawish A. (2024). The interplay of mitochondrial dysfunction in oral diseases: recent updates in pathogenesis and therapeutic implications. Mitochondrion 78, 101942. 10.1016/j.mito.2024.101942 39111357

[B123] Song C. Zhang A. Zhang M. Song Y. Huangfu H. Jin S. (2023). Nrf2/PINK1-mediated mitophagy induction alleviates sodium fluoride-induced hepatic injury by improving mitochondrial function, oxidative stress, and inflammation. Ecotoxicol. Environ. Saf. 252, 114646. 10.1016/j.ecoenv.2023.114646 36791501

[B124] Soto-Heredero G. Gómez de Las Heras M. M. Gabandé-Rodríguez E. Oller J. Mittelbrunn M. (2020). Glycolysis - a key player in the inflammatory response. Febs J. 287 (16), 3350–3369. 10.1111/febs.15327 32255251 PMC7496292

[B125] Su K. K. Yu D. C. Cao X. F. Li P. Chang L. Yu X. L. (2024). Bone marrow mesenchymal stem cell-derived exosomes alleviate nuclear pulposus cells degeneration through the miR-145a-5p/USP31/HIF-1α signaling pathway. Stem Cell Rev. Rep. 20 (8), 2268–2282. 10.1007/s12015-024-10781-9 39212824

[B126] Sun G. Shu T. Ma S. Li M. Qu Z. Li A. (2024a). A submicron forest-like silicon surface promotes bone regeneration by regulating macrophage polarization. Front. Bioeng. Biotechnol. 12, 1356158. 10.3389/fbioe.2024.1356158 38707505 PMC11066256

[B127] Sun H. Zhan M. Zou Y. Ma J. Liang J. Tang G. (2025). Bioactive phosphorus dendrimers deliver protein/drug to tackle osteoarthritis via cooperative macrophage reprogramming. Biomaterials 316, 122999. 10.1016/j.biomaterials.2024.122999 39647219

[B128] Sun H. J. Zheng G. L. Wang Z. C. Liu Y. Bao N. Xiao P. X. (2024b). Chicoric acid ameliorates sepsis-induced cardiomyopathy via regulating macrophage metabolism reprogramming. Phytomedicine 123, 155175. 10.1016/j.phymed.2023.155175 37951150

[B129] Susser L. I. Nguyen M. A. Geoffrion M. Emerton C. Ouimet M. Khacho M. (2023). Mitochondrial fragmentation promotes inflammation resolution responses in macrophages via histone lactylation. Mol. Cell Biol. 43 (10), 531–546. 10.1080/10985549.2023.2253131 37807652 PMC10569354

[B130] Tall A. R. Westerterp M. (2019). Inflammasomes, neutrophil extracellular traps, and cholesterol. J. Lipid Res. 60 (4), 721–727. 10.1194/jlr.S091280 30782961 PMC6446695

[B131] Tannahill G. M. Curtis A. M. Adamik J. Palsson-McDermott E. M. McGettrick A. F. Goel G. (2013). Succinate is an inflammatory signal that induces IL-1β through HIF-1α. Nature 496 (7444), 238–242. 10.1038/nature11986 23535595 PMC4031686

[B132] Teng Y. Huang Y. Yu H. Wu C. Yan Q. Wang Y. (2023). Nimbolide targeting SIRT1 mitigates intervertebral disc degeneration by reprogramming cholesterol metabolism and inhibiting inflammatory signaling. Acta Pharm. Sin. B 13 (5), 2269–2280. 10.1016/j.apsb.2023.02.018 37250166 PMC10213799

[B133] Tian L. Tan Z. Yang Y. Liu S. Yang Q. Tu Y. (2023). *In situ* sprayed hydrogels containing resiquimod-loaded liposomes reduce chronic osteomyelitis recurrence by intracellular bacteria clearance. Acta Biomater. 169, 209–227. 10.1016/j.actbio.2023.07.039 37516419

[B134] Trouillet-Assant S. Gallet M. Nauroy P. Rasigade J. P. Flammier S. Parroche P. (2015). Dual impact of live *Staphylococcus aureus* on the osteoclast lineage, leading to increased bone resorption. J. Infect. Dis. 211 (4), 571–581. 10.1093/infdis/jiu386 25006047

[B135] Van den Bossche J. Baardman J. Otto N. A. van der Velden S. Neele A. E. van den Berg S. M. (2016). Mitochondrial dysfunction prevents repolarization of inflammatory macrophages. Cell Rep. 17 (3), 684–696. 10.1016/j.celrep.2016.09.008 27732846

[B136] Vannella K. M. Wynn T. A. (2017). Mechanisms of organ injury and repair by macrophages. Annu. Rev. Physiol. 79, 593–617. 10.1146/annurev-physiol-022516-034356 27959618

[B137] Viola A. Munari F. Sánchez-Rodríguez R. Scolaro T. Castegna A. (2019). The metabolic signature of macrophage responses. Front. Immunol. 10, 1462. 10.3389/fimmu.2019.01462 31333642 PMC6618143

[B138] Voll R. E. Herrmann M. Roth E. A. Stach C. Kalden J. R. Girkontaite I. (1997). Immunosuppressive effects of apoptotic cells. Nature 390 (6658), 350–351. 10.1038/37022 9389474

[B139] Wang D. K. Zheng H. L. Zhou W. S. Duan Z. W. Jiang S. D. Li B. (2022b). Mitochondrial dysfunction in oxidative stress-mediated intervertebral disc degeneration. Orthop. Surg. 14 (8), 1569–1582. 10.1111/os.13302 35673928 PMC9363752

[B140] Wang H. Lin S. Feng L. Huang B. Lu X. Yang Z. (2023b). Low-Dose staphylococcal enterotoxin C2 mutant maintains bone homeostasis via regulating crosstalk between bone formation and host T-cell effector immunity. Adv. Sci. (Weinh) 10 (28), e2300989. 10.1002/advs.202300989 37552005 PMC10558680

[B141] Wang L. X. Zhang S. X. Wu H. J. Rong X. L. Guo J. (2019). M2b macrophage polarization and its roles in diseases. J. Leukoc. Biol. 106 (2), 345–358. 10.1002/JLB.3RU1018-378RR 30576000 PMC7379745

[B142] Wang W. Liu H. Liu T. Yang H. He F. (2022a). Insights into the role of macrophage polarization in the pathogenesis of osteoporosis. Oxid. Med. Cell Longev. 2022, 2485959. 10.1155/2022/2485959 35707276 PMC9192196

[B143] WangW.WangQ.LiW.XuH.LiangX.WangW. (2024a). Targeting APJ drives BNIP3-PINK1-PARKIN induced mitophagy and improves systemic inflammatory bone loss. J. Adv. Res. 10.1016/j.jare.2024.12.033 39725007

[B144] Wang X. Zhang M. Zhu T. Wei Q. Liu G. Ding J. (2023a). Flourishing antibacterial strategies for osteomyelitis therapy. Adv. Sci. (Weinh) 10 (11), e2206154. 10.1002/advs.202206154 36717275 PMC10104653

[B145] Wang Y. Li N. Zhang X. Horng T. (2021). Mitochondrial metabolism regulates macrophage biology. J. Biol. Chem. 297 (1), 100904. 10.1016/j.jbc.2021.100904 34157289 PMC8294576

[B146] Wang Z. Hu Y. Song J. Ma P. Xia H. (2024b). Polymorphonuclear myeloid-derived suppressor cells regulates immune recovery during HIV infection through PD-L1 and TGF-β pathways. Front. Cell Infect. Microbiol. 14, 1516421. 10.3389/fcimb.2024.1516421 39742336 PMC11685070

[B147] Wang Z. Wang M. Lin M. Wei P. (2024c). The immunomodulatory effects of metformin in LPS-induced macrophages: an *in vitro* study. Inflamm. Res. 73 (2), 175–181. 10.1007/s00011-023-01827-8 38091014

[B148] Weber M. Homm A. Müller S. Frey S. Amann K. Ries J. (2021). Zoledronate causes a systemic shift of macrophage polarization towards M1 *in vivo* . Int. J. Mol. Sci. 22 (3), 1323. 10.3390/ijms22031323 33525753 PMC7865688

[B149] Wen H. Deng H. Li B. Chen J. Zhu J. Zhang X. (2025). Mitochondrial diseases: from molecular mechanisms to therapeutic advances. Signal Transduct. Target Ther. 10 (1), 9. 10.1038/s41392-024-02044-3 39788934 PMC11724432

[B150] Wu C. S. Chen C. Y. Yang C. H. Hsu Y. P. Yu C. H. Chen Y. H. (2025b). The alterations of molecular repertoire of the RANKL-induced osteoclastogenesis in the M1 macrophage-derived inflammatory milieu. Sci. Rep. 15 (1), 16137. 10.1038/s41598-025-99772-5 40341702 PMC12062438

[B151] Wu M. M. Wang Q. M. Huang B. Y. Mai C. T. Wang C. L. Wang T. T. (2021). Dioscin ameliorates murine ulcerative colitis by regulating macrophage polarization. Pharmacol. Res. 172, 105796. 10.1016/j.phrs.2021.105796 34343656

[B152] Wu Q. J. Zhang T. N. Chen H. H. Yu X. F. Lv J. L. Liu Y. Y. (2022a). The sirtuin family in health and disease. Signal Transduct. Target Ther. 7 (1), 402. 10.1038/s41392-022-01257-8 36581622 PMC9797940

[B153] Wu S. K. Wang L. Wang F. Zhang J. (2024a). Resveratrol improved mitochondrial biogenesis by activating SIRT1/PGC-1α signal pathway in SAP. Sci. Rep. 14 (1), 26216. 10.1038/s41598-024-76825-9 39482340 PMC11528064

[B154] Wu X. Chen H. Tian Y. Wang H. Hou H. Hu Q. (2025a). Amelioration of obesity-associated disorders using solanesol with the mitigation of NLRP3 inflammasome activation and macrophage inflammation in adipose tissue. Food Funct. 16 (5), 1903–1918. 10.1039/d4fo05586a 39935386

[B155] Wu X. Xia Y. Dai H. Hong C. Zhao Y. Wei W. (2024b). Metabolic control during macrophage polarization by a citrate-functionalized scaffold for maintaining bone homeostasis. Adv. Healthc. Mater 13 (22), e2400770. 10.1002/adhm.202400770 38626942

[B156] Wu Y. L. Wan S. G. Long Y. Ye H. Yang J. M. Luo Y. (2024c). Correlation between circulating cell-free mitochondrial DNA content and severity of knee degeneration in patients with knee osteoarthritis: a cross-sectional study. Arthritis Res. Ther. 26 (1), 202. 10.1186/s13075-024-03438-y 39558418 PMC11571657

[B157] Wu Y. L. Zhang C. H. Teng Y. Pan Y. Liu N. C. Liu P. X. (2022b). Propionate and butyrate attenuate macrophage pyroptosis and osteoclastogenesis induced by CoCrMo alloy particles. Mil. Med. Res. 9 (1), 46. 10.1186/s40779-022-00404-0 35996168 PMC9396885

[B158] Xia T. Zhang M. Lei W. Yang R. Fu S. Fan Z. (2023). Advances in the role of STAT3 in macrophage polarization. Front. Immunol. 14, 1160719. 10.3389/fimmu.2023.1160719 37081874 PMC10110879

[B159] Xin P. Xu X. Deng C. Liu S. Wang Y. Zhou X. (2020). The role of JAK/STAT signaling pathway and its inhibitors in diseases. Int. Immunopharmacol. 80, 106210. 10.1016/j.intimp.2020.106210 31972425

[B160] Xu W. Sun Y. Wang J. Wang B. Xu F. Xie Z. (2022). Controlled release of silibinin in GelMA hydrogels inhibits inflammation by inducing M2-type macrophage polarization and promotes vascularization *in vitro* . RSC Adv. 12 (21), 13192–13202. 10.1039/d2ra00498d 35520139 PMC9064440

[B161] Xu Z. J. Xia Y. Zhou P. Y. Li J. J. Yang M. G. Zhang Y. (2021). Silicon incorporation into hydroxyapatite nanocarrier counteracts the side effects of vancomycin for efficient chronic osteomyelitis treatment. Chem. Eng. J. 406, 126821. 10.1016/j.cej.2020.126821

[B162] Yan H. Wang Z. Teng D. Chen X. Zhu Z. Chen H. (2024). Hexokinase 2 senses fructose in tumor-associated macrophages to promote colorectal cancer growth. Cell Metab. 36 (11), 2449–67.e6. 10.1016/j.cmet.2024.10.002 39471815

[B163] Yang H. Wang Y. Zhen S. Wang B. Jiao M. Liu L. (2023). AMPK activation attenuates cancer-induced bone pain by reducing mitochondrial dysfunction-mediated neuroinflammation. Acta Biochim. Biophys. Sin. (Shanghai) 55 (3), 460–471. 10.3724/abbs.2023039 36971458 PMC10160234

[B164] Yang T. Liu S. Ma H. Lai H. Wang C. Ni K. (2024a). Carnitine functions as an enhancer of NRF2 to inhibit osteoclastogenesis via regulating macrophage polarization in osteoporosis. Free Radic. Biol. Med. 213, 174–189. 10.1016/j.freeradbiomed.2024.01.017 38246515

[B165] Yang W. Li K. Pan Q. Huang W. Xiao Y. Lin H. (2024b). An engineered bionic nanoparticle sponge as a cytokine trap and reactive oxygen species scavenger to relieve disc degeneration and discogenic pain. ACS Nano 18 (4), 3053–3072. 10.1021/acsnano.3c08097 38237054 PMC10832058

[B166] Yang X. Xu S. Qian Y. Xiao Q. (2017). Resveratrol regulates microglia M1/M2 polarization via PGC-1α in conditions of neuroinflammatory injury. Brain Behav. Immun. 64, 162–172. 10.1016/j.bbi.2017.03.003 28268115

[B167] Yang Y. Zhou H. Li F. Zhang Y. Yang J. Shen Y. (2025). *Staphylococcus aureus* induces mitophagy via the HDAC11/IL10 pathway to sustain intracellular survival. J. Transl. Med. 23 (1), 156. 10.1186/s12967-025-06161-7 39905391 PMC11796158

[B168] Zhang D. Tang Z. Huang H. Zhou G. Cui C. Weng Y. (2019). Metabolic regulation of gene expression by histone lactylation. Nature 574 (7779), 575–580. 10.1038/s41586-019-1678-1 31645732 PMC6818755

[B169] Zhang D. Wu Y. Li Z. Chen H. Huang S. Jian C. (2021). MiR-144-5p, an exosomal miRNA from bone marrow-derived macrophage in type 2 diabetes, impairs bone fracture healing via targeting Smad1. J. Nanobiotechnology 19 (1), 226. 10.1186/s12951-021-00964-8 34340698 PMC8327443

[B170] Zhang D. Yang X. Y. Qin Y. Z. Wu G. D. Ning G. B. Huo N. R. (2020a). Antagonistic effect of N-acetyl-L-cysteine against cadmium-induced cytotoxicity and abnormal immune response on chicken peritoneal macrophages. Ecotoxicol. Environ. Saf. 206, 111185. 10.1016/j.ecoenv.2020.111185 32890923

[B171] Zhang F. Zhang Y. Zhou J. Cai Y. Li Z. Sun J. (2024a). Metabolic effects of quercetin on inflammatory and autoimmune responses in rheumatoid arthritis are mediated through the inhibition of JAK1/STAT3/HIF-1α signaling. Mol. Med. 30 (1), 170. 10.1186/s10020-024-00929-1 39390367 PMC11468292

[B172] Zhang H. Cai D. Bai X. (2020b). Macrophages regulate the progression of osteoarthritis. Osteoarthr. Cartil. 28 (5), 555–561. 10.1016/j.joca.2020.01.007 31982565

[B173] Zhang L. Gan X. He Y. Zhu Z. Zhu J. Yu H. (2017). Drp1-dependent mitochondrial fission mediates osteogenic dysfunction in inflammation through elevated production of reactive oxygen species. PLoS One 12 (4), e0175262. 10.1371/journal.pone.0175262 28388678 PMC5384744

[B174] Zhang M. Wu J. Cai K. Liu Y. Lu B. Zhang J. (2024e). From dysfunction to healing: advances in mitochondrial therapy for Osteoarthritis. J. Transl. Med. 22 (1), 1013. 10.1186/s12967-024-05799-z 39529128 PMC11552139

[B175] Zhang S. Hou B. Xu A. Wen Y. Zhu X. Cai W. (2024d). Ganlu formula ethyl acetate extract (GLEE) blocked the development of experimental arthritis by inhibiting NLRP3 activation and reducing M1 type macrophage polarization. J. Ethnopharmacol. 332, 118377. 10.1016/j.jep.2024.118377 38782307

[B176] Zhang S. Wang D. Ding Y. Li Y. Wang Y. Zeng J. (2024b). Inhibition of calpain reduces oxidative stress and attenuates pyroptosis and ferroptosis in *Clostridium perfringens* Beta-1 toxin-induced macrophages. Microbiol. Res. 289, 127916. 10.1016/j.micres.2024.127916 39342748

[B177] Zhang W. Lu H. Zhang W. Hu J. Zeng Y. Hu H. (2024c). Inflammatory microenvironment-responsive hydrogels enclosed with *quorum* sensing inhibitor for treating post-traumatic osteomyelitis. Adv. Sci. (Weinh) 11 (20), e2307969. 10.1002/advs.202307969 38482752 PMC11132068

[B178] Zhang Y. Ji Q. (2023). Macrophage polarization in osteoarthritis progression: a promising therapeutic target. Front. Cell Dev. Biol. 11, 1269724. 10.3389/fcell.2023.1269724 37954210 PMC10639142

[B179] Zhang Y. Liu Y. Hou M. Xia X. Liu J. Xu Y. (2023c). Reprogramming of mitochondrial respiratory chain complex by targeting SIRT3-COX4I2 Axis attenuates osteoarthritis progression. Adv. Sci. (Weinh) 10 (10), e2206144. 10.1002/advs.202206144 36683245 PMC10074136

[B180] Zhang Y. Zhang L. Zhao Y. He J. Zhang Y. Zhang X. (2023a). PGC-1α inhibits M2 macrophage polarization and alleviates liver fibrosis following hepatic ischemia reperfusion injury. Cell Death Discov. 9 (1), 337. 10.1038/s41420-023-01636-2 37679346 PMC10484946

[B181] Zhang Z. Xie S. Qian J. Gao F. Jin W. Wang L. (2023b). Targeting macrophagic PIM-1 alleviates osteoarthritis by inhibiting NLRP3 inflammasome activation via suppressing mitochondrial ROS/Cl(-) efflux signaling pathway. J. Transl. Med. 21 (1), 452. 10.1186/s12967-023-04313-1 37422640 PMC10329339

[B182] Zhao X. Wang Q. Wang W. Lu S. (2024). Increased neutrophil extracellular traps caused by diet-induced obesity delay fracture healing. Faseb J. 38 (20), e70126. 10.1096/fj.202401523R 39446097 PMC11580727

[B183] Zhao Z. Ming Y. Li X. Tan H. He X. Yang L. (2023). Hyperglycemia aggravates periodontitis via autophagy impairment and ROS-inflammasome-mediated macrophage pyroptosis. Int. J. Mol. Sci. 24 (7), 6309. 10.3390/ijms24076309 37047282 PMC10094233

[B184] Zheng Y. Wei K. Jiang P. Zhao J. Shan Y. Shi Y. (2024). Macrophage polarization in rheumatoid arthritis: signaling pathways, metabolic reprogramming, and crosstalk with synovial fibroblasts. Front. Immunol. 15, 1394108. 10.3389/fimmu.2024.1394108 38799455 PMC11116671

[B185] Zhu D. Chen F. Qiang H. Qi H. (2024a). SPA inhibits hBMSC osteogenic differentiation and M1 macrophage polarization by suppressing SETD2 in acute suppurative osteomyelitis. Sci. Rep. 14 (1), 12728. 10.1038/s41598-024-63219-0 38830934 PMC11148074

[B186] Zhu X. X. Zheng G. L. Lu Q. B. Su J. B. Liu Y. Wang M. (2024b). Cichoric acid ameliorates sepsis-induced acute kidney injury by inhibiting M1 macrophage polarization. Eur. J. Pharmacol. 976, 176696. 10.1016/j.ejphar.2024.176696 38821160

[B187] Zhu Y. Chen X. Lu Y. Xia L. Fan S. Huang Q. (2022). Glutamine mitigates murine burn sepsis by supporting macrophage M2 polarization through repressing the SIRT5-mediated desuccinylation of pyruvate dehydrogenase. Burns Trauma 10, tkac041. 10.1093/burnst/tkac041 36601059 PMC9801296

[B188] Zuo S. Wang Y. Bao H. Zhang Z. Yang N. Jia M. (2024). Lipid synthesis, triggered by PPARγ T166 dephosphorylation, sustains reparative function of macrophages during tissue repair. Nat. Commun. 15 (1), 7269. 10.1038/s41467-024-51736-5 39179603 PMC11343878

